# Chemical Genomics Identifies the PERK-Mediated Unfolded Protein Stress Response as a Cellular Target for Influenza Virus Inhibition

**DOI:** 10.1128/mBio.00085-16

**Published:** 2016-04-19

**Authors:** Sara Landeras-Bueno, Yolanda Fernández, Ana Falcón, Juan Carlos Oliveros, Juan Ortín

**Affiliations:** a Departamento de Biología Molecular y Celular, Centro Nacional de Biotecnología (CSIC), Madrid, Spain; b Ciber de Enfermedades Respiratorias (ISCIII), Madrid, Spain; c Servicio de Genómica Computacional, Centro Nacional de Biotecnología (CSIC), Madrid, Spain

## Abstract

Influenza A viruses generate annual epidemics and occasional pandemics of respiratory disease with important consequences for human health and the economy. Therefore, a large effort has been devoted to the development of new anti-influenza virus drugs directed to viral targets, as well as to the identification of cellular targets amenable to anti-influenza virus therapy. Here we have addressed the identification of such potential cellular targets by screening collections of drugs approved for human use. We reasoned that screening with a green fluorescent protein-based recombinant replicon system would identify cellular targets involved in virus transcription/replication and/or gene expression and hence address an early stage of virus infection. By using such a strategy, we identified Montelukast (MK) as an inhibitor of virus multiplication. MK inhibited virus gene expression but did not alter viral RNA synthesis *in vitro* or viral RNA accumulation *in vivo*. The low selectivity index of MK prevented its use as an antiviral, but it was sufficient to identify a new cellular pathway suitable for anti-influenza virus intervention. By deep sequencing of RNA isolated from mock- and virus-infected human cells, treated with MK or left untreated, we showed that it stimulates the PERK-mediated unfolded protein stress response. The phosphorylation of PERK was partly inhibited in virus-infected cells but stimulated in MK-treated cells. Accordingly, pharmacological inhibition of PERK phosphorylation led to increased viral gene expression, while inhibition of PERK phosphatase reduced viral protein synthesis. These results suggest the PERK-mediated unfolded protein response as a potential cellular target to modulate influenza virus infection.

## IMPORTANCE

Influenza A viruses are responsible for annual epidemics and occasional pandemics with important consequences for human health and the economy. The unfolded protein response is a defense mechanism fired by cells when the demand of protein synthesis and folding is excessive, for instance, during an acute virus infection. In this report, we show that influenza virus downregulates the unfolded protein response mediated by the PERK sensor, while Montelukast, a drug used to treat asthma in humans, specifically stimulated this response and downregulated viral protein synthesis and multiplication. Accordingly, we show that PERK phosphorylation was reduced in virus-infected cells and increased in cells treated with Montelukast. Hence, our studies suggest that modulation of the PERK-mediated unfolded protein response is a target for influenza virus inhibition.

## INTRODUCTION

Influenza A viruses are the causative agents of annual epidemics of respiratory disease and occasionally generate pandemics with variable consequences for human health and the global economy (1). The 1918 pandemic caused some 50 million deaths (2), while the last pandemic, in 2009, was much milder and a normal yearly epidemic causes up to 500,000 deaths worldwide (http://www.who.int/mediacentre/factsheets/fs211/en/).

The genome of influenza A viruses is a set of eight single-stranded, negative-polarity RNAs that are assembled in ribonucleoprotein complexes (RNPs) and incorporated into enveloped particles (3). Upon entry into the infected cell, viral RNPs are imported into the nucleus, where viral transcription and replication take place. The first step in virus gene expression is transcription from the parental RNPs, and translation of these early viral mRNAs is essential for viral RNP replication (4). At least the virus nucleoprotein (NP) and polymerase (PB1, PB2, and PA proteins) are necessary to produce progeny RNPs (5). This process involves first the generation of complementary RNPs that serve as efficient templates for the production of large amounts of progeny RNPs (reviewed in references 6 to 9). Progeny RNPs are then exported back to the cytoplasm and bud from the cell membrane (reviewed in reference 10).

At present, two types of anti-influenza virus drugs are available, the adamantanes, which block the M2 ion channel, and the neuraminidase inhibitors, which interfere with virion release (reviewed in reference 11), as well as the polymerase inhibitor favipiravir (T-705) (12), which has been recently approved in Japan. In addition, a number of other compounds have been described that are specific for targets related to virus transcription/replication and may become useful in the clinic in the future (13–21) and others have been reported to inhibit virus-induced membrane fusion (reviewed in reference 22). However, a common problem for the virus target-directed antivirals is the generation of resistant virus strains. Most circulating influenza viruses are resistant to adamantanes, and virus strains resistant to neuraminidase inhibitors have been described upon clinical use (23) or independently of drug use (24). Likewise, some of the potential new inhibitors have been shown to readily select for resistant mutants (16, 21). Hence, a large effort has been made to identify cellular targets that could be useful in indirectly reducing influenza virus multiplication and/or pathogenesis.

Early efforts in this direction came from the observation that some signaling pathways are altered during influenza virus infection (25–29), and modulation of influenza virus multiplication was attempted with drugs designed to alter cell signaling (reviewed in references 30 and 31). These approaches reflect the general requirement of cellular pathways and biosynthetic machineries for virus multiplication that has been the subject of much attention in recent years. Thus, the identification of cellular factors important for influenza virus replication has been addressed by a number of different approaches, like targeted (32–34) or general (35) two-hybrid screens, as well as targeted proteomic analyses of virus-containing intracellular complexes (36, 37) or purified virions (38, 39). In addition, genome-wide downregulation of cellular gene expression has identified a plethora of genes that can alter influenza virus multiplication (35, 40–43; reviewed in reference 44). This information has been complemented with general analyses of the alterations in cellular gene expression induced by influenza virus infection, at either the RNA (45–47) or the protein (48, 49) level. Knowledge derived from these virus-host interaction studies has fostered the search for inhibitors targeted to cellular factors that may serve as influenza antivirals (reviewed in references 50 to 53).

Here we have taken an alternative approach to identify potential cellular targets for anti-influenza virus intervention. By screening collections of drugs approved for human use in a green fluorescent protein (GFP)-based virus transcription-replication system, we identified Montelukast (MK) as an inhibitor of virus gene expression and validated these results in virus-infected cells. The selectivity index of MK was rather low, and hence, it is not a potential antiviral *per se*, but we used it as a probe to identify the cellular target affected. By deep sequencing of RNA isolated from mock- and virus-infected human cells, treated with MK or left untreated, we showed that it counteracts the influenza virus-induced block in the PERK-mediated unfolded protein stress response. In agreement with these alterations in cellular gene expression, PERK phosphorylation was inhibited in infected cells but stimulated in MK-treated cells, and accordingly, inhibition of PERK phosphorylation led to increased viral gene expression. These results suggest the PERK-mediated unfolded protein response as a potential cellular target to modulate influenza virus infection.

## RESULTS AND DISCUSSION

### Screening of clinically tested human drugs for influenza virus inhibition in a GFP-based replicon assay.

With the aim to identify new cellular targets for influenza virus inhibition, we reasoned that screening with a recombinant replicon system would address an early step in the virus replication cycle, namely, virus transcription/replication and/or gene expression, and hence provide a stage of virus infection more useful for inhibition. In addition, we reasoned that by using a restricted library of compounds that have been approved as drugs for human use would most probably ensure that a cellular target would be addressed.

**FIG 1:**
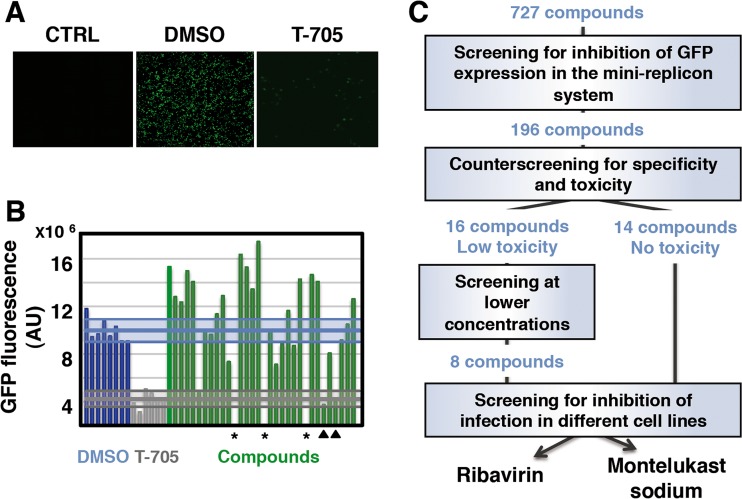
Recombinant GFP-based screening for influenza virus inhibitors. Cultures of human HEK293T cells in 96-well plates were used to reconstitute a GFP minireplicon system and treated with vehicle (DMSO) or favipiravir (T-705). As a control, similar experiments were performed without the PB1 subunit of the viral polymerase (CTRL). (A) Fluorescence images. (B) GFP fluorescence values of DMSO-treated wells (blue), T-705-treated wells (gray), and individual wells treated with specific compounds from the NIH library (green). The blue and gray horizontal bars indicate the averages of DMSO- or T-705-treated wells, and the corresponding shadowed zones indicate the standard deviations. Specific compounds that are potentially toxic (stars) or inhibitors of GFP expression (triangles) were identified. AU, arbitrary units. (C) Overview of the screening process. Out of 727 specific compounds from the NIH libraries, 495 did not alter the GFP signal, 36 compounds increased the GFP signal, and 196 decreased the GFP signal. These 196 compounds were used for counterscreening for toxicity and specificity. Cultures of human HEK293T cells in 96-well plates were transfected with plasmid pCAGGsGFP or used to reconstitute RNP-GFP and treated individually with the 196 candidate compounds. Fourteen compounds showed no toxicity, and 16 were partly toxic and were tested at lower concentrations. Finally, 22 compounds decreased the GFP signal in the context of the replicon system (RNP-GFP) but not in the context of an RNA polymerase II-dependent system (pCAGGsGFP). These 22 compounds were used for inhibition of infection of A549 and Huh7 cells with the VIC and WSN strains. Only ribavirin, a known inhibitor of influenza virus infection, and MK reduced virus yields under all of the conditions analyzed.

To set up a screening platform, we adapted our previous know-how with viral replicon systems expressing chloramphenicol acetyltransferase to use a more versatile GFP marker. Recombinant RNPs containing the GFP-encoding gene in negative polarity were generated by transfection of viral RNA polymerase, NP, and genomic plasmids into human HEK293T cells as previously described (54). As a negative control, RNPs were prepared from which the plasmid encoding the PB1 subunit of the polymerase was omitted. In addition, we downscaled it to a 96-well plate format to carry out medium-throughput screening. After optimizing the transfection parameters and replication time, we could observe reproducible GFP expression in most of the cells in each culture (Fig. 1A, DMSO [dimethyl sulfoxide]) and a very low background fluorescence level in the negative-control cultures (Fig. 1A, CTRL). To determine the quality of the screening system, multiple transfected cultures were analyzed for fluorescence signal, standard deviation, reproducibility factor, coefficient of variation, and signal-to-noise and signal-to-background ratios (55). The results are shown in Fig. S1 in the supplemental material, and the Z factor obtained indicates that the screening system developed is a fully suited assay. As a positive control for the inhibition of reporter expression, we used favipiravir (T-705), a well-known inhibitor of virus RNA replication (12). As presented in Fig. 1A (T-705) and quantitated in Fig. 1B, statistically significant inhibition was observed upon the addition of favipiravir 2 h after transfection (*P* < 0.0001, as determined by the Student *t* test).

We then used NIH libraries of approved drugs, including a total of 727 compounds, to perform screening in the system, always including negative (DMSO) and positive (T-705) controls in the same plate. We chose a dose of 50 µM for the first screening, as pilot tests indicated that less than 10% of the compounds led to toxicity or inhibition. A sample of the results obtained is presented in Fig. 1B. Most of the compounds were not active, but some of them abolished GFP expression (Fig. 1B, stars). Another group of compounds (*n* = 196) reduced GFP to levels similar to those obtained with T-705 (Fig. 1B, arrowheads) and were selected for further analyses (Fig. 1C). To verify their activity and rule out unspecific or toxic compounds, the screening was repeated with both the indicated GFP-based replicon system and a control system in which GFP expression was driven by a polymerase II promoter (i.e., was independent of any virus element). The compounds that showed potential toxicity (i.e., induced aberrant cell morphology or reduced the cellular mass of the cultures) were further tested at lower concentrations (3, 6, 12, and 25 µM). In this way, 22 compounds were selected that showed activity in the GFP-based replicon system but did not inhibit cellular GFP expression (Fig. 1C). Finally, these 22 compounds were tested for inhibition of virus replication in low-multiplicity infections with the VIC (H3N2) and WSN (H1N1) strains of influenza virus in two human cell lines, A549 and Huh7, of epithelial and hepatic origins, respectively. Two compounds were found to reproducibly reduce virus yields: ribavirin, a known wide-range virus inhibitor (56), and MK sodium (see Fig. S2A in the supplemental material). To verify that MK was indeed able to reduce virus titers, the compound was purchased from a different provider, the identities of both the NIH Collection and the alternative compound were verified by mass spectrometry (see Fig. S2B and C) and the inhibition of virus multiplication was again verified by low-multiplicity infections as described above.

**FIG 2:**
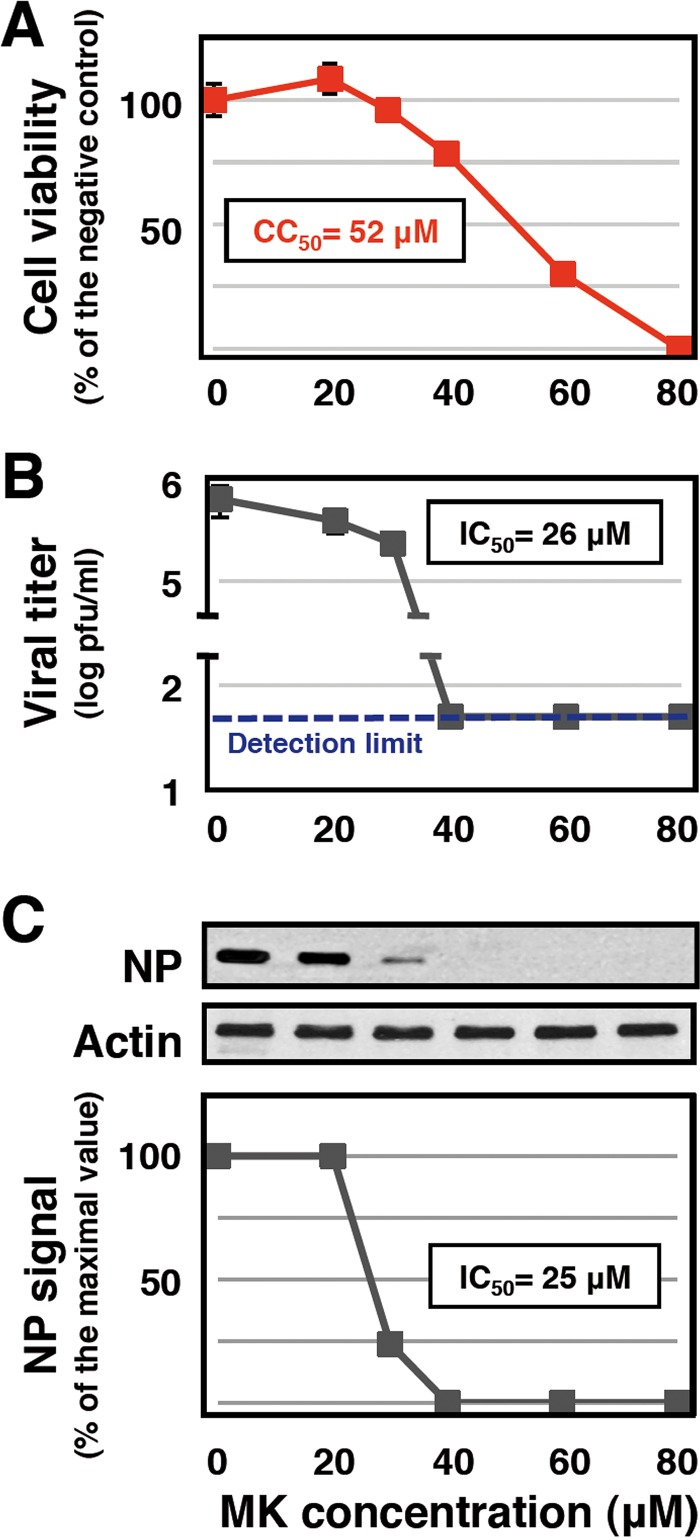
Efficacy and toxicity of MK during influenza virus infection. (A) Cultures of human A549 cells were treated with the concentrations of MK indicated, and the mitochondrial activity of the cells was measured at 48 hpi with the MTT assay. (B) Cultures of human A549 cells were infected with the VIC virus strain at an MOI of 0.001 PFU/cell and treated with the concentrations of MK indicated. Virus titration was performed with samples obtained at 48 hpi. (C) Cultures of human A549 cells were infected with VIC at an MOI of 3 PFU/cell and treated with the concentrations of MK indicated. At 6 hpi, the accumulation of NP and β-actin was determined by Western blotting with specific antibodies. The quantification of the NP signal is represented at the bottom. The data in panels A and B are presented as averages and standard deviations of three independent biological replicates.

To evaluate the toxicity and effectiveness of MK, cultures of A549 human cells were treated with a range of compound concentrations and cell viability was determined by the 3-(4,5-dimethyl-2-thiazolyl)-2,5-diphenyl-2H-tetrazolium bromide (MTT) assay 48 h later. The results are presented in Fig. 2A and indicated a 50% cytotoxic concentration (CC_50_) of 52 ± 2 µM. Parallel cultures were treated under the same conditions and infected with the VIC influenza virus strain at 0.001 PFU/cell. At 48 h postinfection (hpi), virus titers in the supernatants were determined by plaque assay in MDCK cells. The results are presented in Fig. 2B and indicated a 50% inhibitory concentration (IC_50_) of around 26 µM. It is important to mention that virus production in the presence of 40 µM MK was below the limit of detection (Fig. 2B), in spite of only a marginal reduction in cell viability (Fig. 2A). To verify these results, cultures of A549 human cells were infected with the VIC influenza virus strain at 3 PFU/cell and treated with various MK concentrations and total cell extracts were prepared at 6 hpi. Under these conditions, no apparent cytotoxic effect on mock-infected cells was observed at any of the MK concentration used (data not shown) but the accumulation of viral NP showed a progressive decline, as determined by Western blotting, with an estimated IC_50_ of around 25 µM (Fig. 2C). These results indicate that MK is able to reduce influenza virus yields and reduces viral gene expression at nontoxic concentrations, in good agreement with the design of the screening procedure used, but indicates a selectivity index of around 2. Such a low selectivity index precludes the use of MK as a potential influenza virus inhibitor but still allows further studies to identify the target affected.

### MK preferentially inhibits viral protein synthesis.

**FIG 3:**
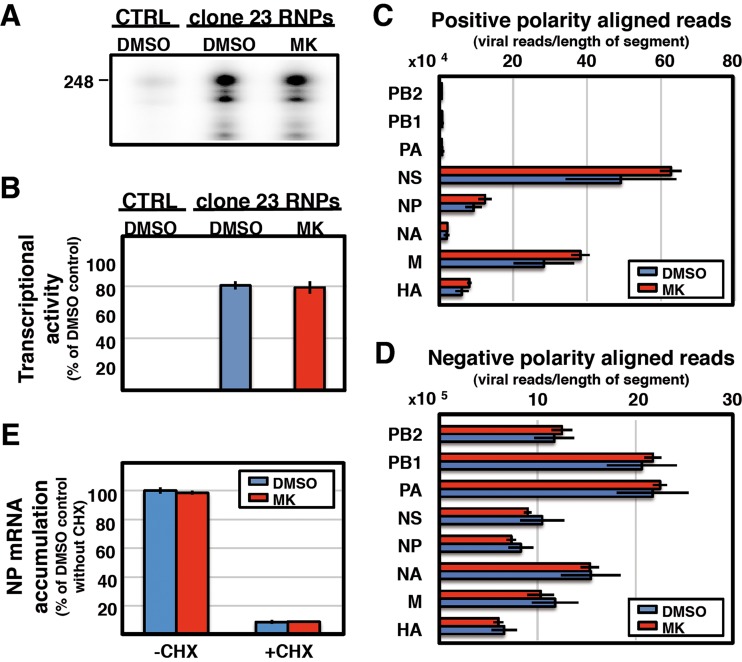
MK treatment does not alter influenza virus RNA synthesis. (A) Cultures of human HEK293T cells were transfected with plasmids expressing the polymerase subunits and the NP, as well as with a genomic plasmid expressing a deleted NS RNA segment (clone 23) (57) in negative polarity. As a control, similar experiments were performed without the PB1 subunit of the viral polymerase (CTRL). Extracts of these cultures were used for *in vitro* transcription with β-globin mRNA as a cap donor and 20 µM MK or the corresponding amount of DMSO. The transcripts were analyzed by denaturing polyacrylamide gel electrophoresis. The mobility of a genome-size marker of 248 nt is indicated to the left. (B) Quantification of the results in panel A as percentages of the transcriptional activity of RNPs treated with DMSO. Average values and standard deviation of three independent experiments are presented. (C, D) Cultures of human A549 cells were infected with influenza virus at an MOI of 3 PFU/cell and then treated with 20 µM MK or the corresponding amount of DMSO. Total cell RNA was isolated at 6 hpi, and rRNA was removed. Viral RNA of each segment was determined by deep sequencing and classified according to the virus segment and polarity. The results were standardized by the total number of reads per sample. The use of total histone mRNA reads for standardization led to similar results. (C) Total accumulation of positive-polarity RNA of each viral segment. (D) Total accumulation of negative-polarity RNA of each viral segment. The data in panels C and D are presented as averages and standard deviations of three independent biological replicates. (E) Cultures of human A549 cells were infected with influenza virus at an MOI of 3 PFU/cell and then treated with 20 µM MK or the corresponding amount of DMSO in the presence or absence of cycloheximide (100 µg/ml). Total cell RNA was isolated at 6 hpi, and the accumulation of viral RNA was determined by RT-qPCR with probes specific for the NP segment. The accumulation of viral mRNA in cells treated with cycloheximide (CHX) and/or MK or not treated is presented as the average and standard deviation of three determinations.

**FIG 4:**
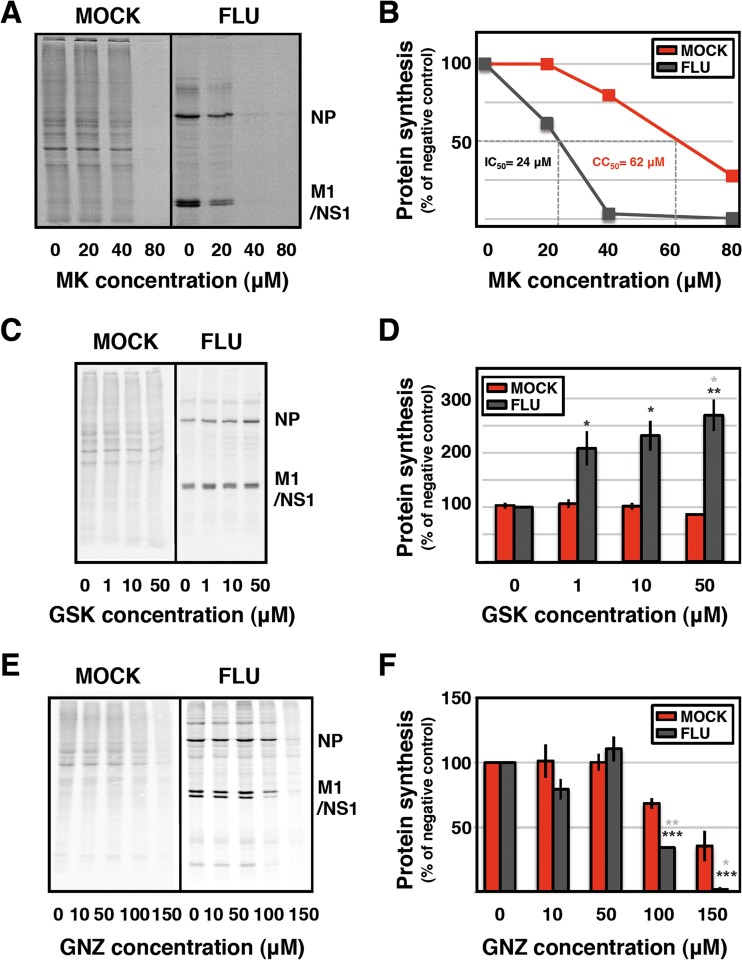
MK treatment preferentially inhibits influenza virus-specific protein synthesis. Cultures of human A549 cells were infected with influenza virus strain VIC (FLU) at an MOI of 1 to 3 PFU/cell and treated with the concentrations of MK (A and B), GSK-2656157 (GSK) (C, D), or guanabenz (GNZ) (E and F) indicated. At 6 hpi, the cultures were pulse-labeled with [^35^S]methionine-cysteine and total protein extracts were prepared. The samples were analyzed by polyacrylamide gel electrophoresis and autoradiography. (A, C, E) Autoradiographs of polyacrylamide electrophoresis gels from representative experiments are presented. The mobility of some of the virus-specific proteins is indicated to the right. (B, D, F) Quantification of the signals of cellular proteins (red) and viral proteins (gray) at the MK, GSK, or GNZ concentrations indicated. The average and standard deviation of three independent experiments are presented. Significance of treated-infected versus nontreated-infected cells (black stars) or mock-infected versus infected cells (gray stars) was determined by the Student *t* test (*, *P* < 0.05; **, *P* < 0.01; ***, *P* < 0.001).

To determine whether the reduction of virus protein accumulation observed under treatment with MK was due to an inhibition of virus transcription/replication or viral protein synthesis, we first measured the *in vitro* activity of recombinant RNPs treated with MK or left untreated. Recombinant mini-RNPs containing a 248-nucleotide (nt)-long template (clone 23 RNPs) were generated by transfection of viral polymerase, NP, and genomic plasmids into human HEK293T cells as previously described (57). As a negative control, parallel transfections were carried out in which the plasmid encoding the PB1 subunit of the polymerase was omitted. The RNA synthesis capacity of total cell extracts was determined in the presence of 20 µM MK or the corresponding amount of vehicle (DMSO) by quantification of the transcript generated, after separation by denaturing gel electrophoresis. An example of the results is presented in Fig. 3A, and the quantitative data of several replicate experiments are shown in Fig. 3B. These results indicate that MK does not alter viral RNA synthesis *in vitro*. To analyze viral RNA synthesis during infection, we carried out deep sequencing of virus-specific RNAs present in mock- or virus-infected human cells treated with MK or left untreated. We chose 6 hpi as the time point because viral transcription and replication are well under way in cells infected at a high multiplicity (3 PFU/cell) and a 20 µM concentration of MK because it is not toxic to the cells and leads to partial viral protein synthesis inhibition (see Fig. 4). Duplicate biological replicates were subjected to Illumina sequencing after removal of rRNA. Analysis of the virus RNA sequences obtained verified that treatment with MK does not alter the accumulation of positive-polarity or negative-polarity viral RNAs during infection (Fig. 3C and D) and also indicated that the virus splicing process is unaltered by MK, since the M1/M2 and NS1/NEP mRNA ratios are indistinguishable under both experimental conditions (see Fig. S3 in the supplemental material). To further verify whether viral transcription is altered by MK treatment, the accumulation of primary transcripts was determined by RT-qPCR of total cell RNA isolated from cells infected in the presence of cycloheximide, treated with MK or left untreated. The results are presented in Fig. 3E and confirm that MK does not affect influenza virus transcription.

Next, we determined the effect of MK on the protein synthesis activity in mock- or virus-infected cells. Cultures of A549 cells were infected at 3 PFU/cell or mock infected and treated with various amounts of MK. At 6 hpi, the cells were labeled with [^35^S]methionine-cysteine for 1 h and total cell extracts were prepared and analyzed by denaturing gel electrophoresis and autoradiography. The results of a representative experiment are shown in Fig. 4A and indicate partial inhibition of viral protein synthesis at 20 µM MK and almost complete inhibition at 40 µM. In contrast, cellular protein synthesis was almost unaltered at 20 µM MK and only marginally affected at 40 µM MK. These data result in a CC_50_ of about 62 µM and an IC_50_ of around 24 µM (Fig. 4B), in good agreement with previous results (Fig. 2), and suggest that viral protein synthesis is the target of MK action during infection, although nucleocytoplasmic mRNA transport could not be excluded at this point (but see below).

### MK stimulates the PERK-dependent pathway of the unfolded protein response.

**FIG 5:**
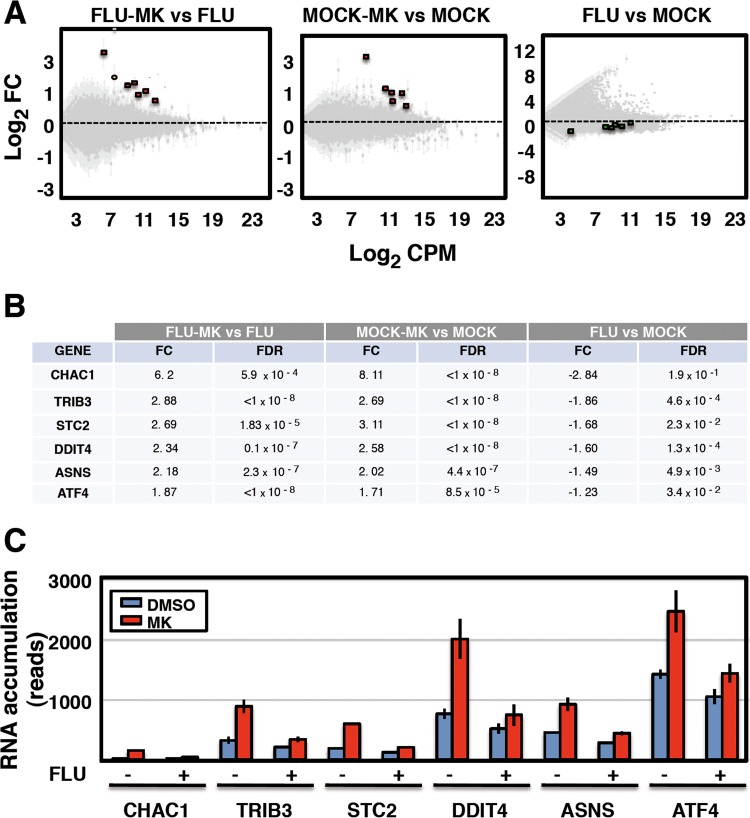
The PERK-dependent unfolded protein response pathway is differentially altered by influenza virus infection or MK treatment. Cultures of A549 cells were infected with influenza virus at an MOI of 3 (FLU) or mock infected (MOCK) and treated with 20 µM MK or not treated. At 6 hpi, total nonribosomal RNA was subjected to RNA-Seq analysis. The results were standardized by the total number of reads per sample. The use of total histone mRNA reads for standardization led to similar results. (A) FIESTA diagrams (97) indicating the number of differentially expressed human genes across the comparison of MK-treated versus DMSO-treated infected cells (left side), MK-treated versus untreated mock-infected cells (middle), and infected versus mock-infected cells (right side). On the left side, the genes with an FDR of <10^−3^ and an FC of >1.7 are shown with red dots. The yellow dot corresponds to a pseudogene with an FDR of >5.10^−3^. The position of this set of genes in the other comparisons is marked in red (middle) or green (right side). (B) Table with the products of the genes marked with red dots on the left side of panel A. The FC and FDR are presented for each of the comparisons shown in panel A. (C) The accumulation of RNA for the genes listed in panel B, obtained from DMSO-treated (blue), MK-treated (red), mock-infected (−), or influenza virus-infected (+) cells by deep sequencing, is shown (number of reads standardized as in Fig. 3D). The data are presented as the average and range of two independent biological replicates.

As neither *in vitro* transcription of viral RNPs nor accumulation of viral RNAs in infected cells was altered by MK addition (Fig. 3), we assumed that the target of MK anti-influenza virus activity is cellular. To identify such a potential cellular target, we carried out deep-sequencing analysis of the cellular RNAs present in mock- or virus-infected human cells treated with MK or left untreated. Duplicate cultures of A549 cells were infected at 3 PFU/cell or mock infected and treated with 20 µM MK, a dose that neither affected cell viability (Fig. 2A) nor reduced cellular protein synthesis (Fig. 4A), or left untreated. Deep-sequencing analysis was started by comparing the expression of cellular RNAs in virus-infected cells treated with MK or left untreated. When a false-discovery rate (FDR) of <10^−3^ was used as a statistical significance cutoff, a number of cellular genes were overexpressed in virus-infected cells upon MK treatment with changes ranging from 1.87- to 6.2-fold (Fig. 5A, left side, red dots, and B). The potential interactions between the cellular genes identified in Fig. 5 were tested by using the STRING database, and several of them (ATF4, TRIB3, DDIT4, and ASNS) appeared to be connected in an interaction network, based on experimental, text mining, and database searching evidence (see Fig. S4 in the supplemental material, where they are circled in red), and they all represent genes downstream of the PERK-mediated arm of the unfolded protein stress response (UPR) (58, 59). To verify that the ATF6 and IRE1 arms of the UPR were also induced by MK treatment, the RNA accumulation of a number of their downstream genes was checked and none was upregulated in virus-infected cells upon MK treatment (see Table S1 in the supplemental material; FLU-MK versus FLU). Thus, it could be concluded that MK specifically stimulates the PERK arm of the UPR during virus infection.

When the gene set described in Fig. 5B was considered in the two alternative comparisons included in the experimental setting, it became clear that they were all overexpressed upon MK treatment of mock-infected cells (Fig. 5A, middle, red dots, and B) and they were all downregulated in virus-infected versus mock-infected cells, albeit to a lesser extent (Fig. 5A, right side, green dots, and B). For a direct comparison, these data are jointly represented in Fig. 5C and all together indicate that MK treatment is able to restore the expression of this set of genes to the normal levels observed in untreated cells. To test whether MK treatment counteracts the downregulation of any gene as a consequence of virus infection, we selected those showing a fold change (FC) of less than −3 and an FDR of <10^−3^. The alterations of the 15 genes identified upon MK treatment were analyzed, and the results indicate that MK treatment does not unspecifically counteract cellular genes downregulated by virus infection (see Table S2 in the supplemental material).

### MK induces PERK phosphorylation and counteracts the influenza virus-induced block of the PERK pathway.

**FIG 6:**
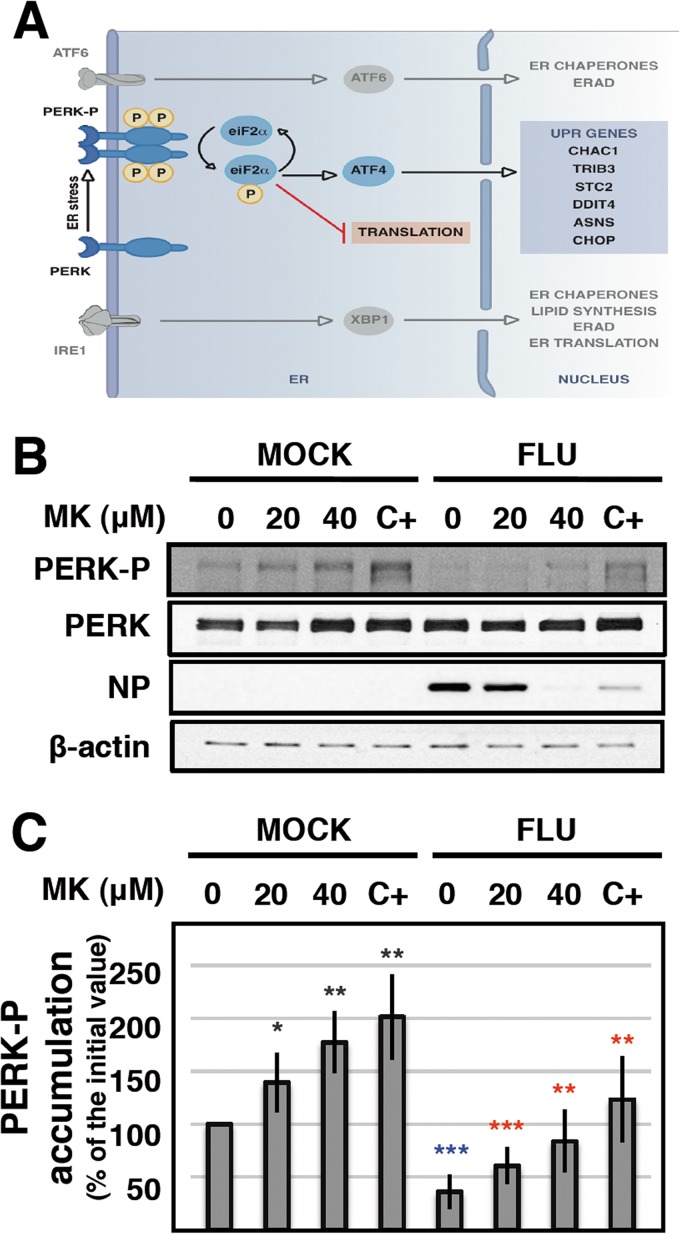
MK treatment reverses influenza virus-induced PERK dephosphorylation. (A) Scheme of pathways activated by accumulation of unfolded or misfolded proteins in the lumen of the endoplasmic reticulum. The pathways activated by ATF6, PERK, and IRE1 are indicated. Most relevant for this study, the PERK-dependent pathway is highlighted, including the eiF2α-dependent translation block and the downstream genes identified here as upregulated by MK treatment in virus-infected cells. (B) Cultures of human A549 cells were infected with VIC virus at an MOI of 3 PFU/cell (FLU) or mock-infected (MOCK) and treated with 20 or 40 µM MK or 1 mM of thapsigargin (C+). At 6 hpi, the accumulation of P-PERK, total PERK, NP, and β-actin was determined by Western blotting with specific antibodies. (C) The quantification of the P-PERK signal is presented as the average and standard deviation of five determinations. Significance of differences between treated cells and untreated mock-infected cells (black stars), between treated and untreated virus-infected cells (red stars), and between infected cells and mock-infected cells without treatment (blue stars) was determined by the Student *t* test (*, *P* < 0.05; **, *P* < 0.01; ***, *P* < 0.001).

The first step in the induction of the PERK-dependent UPR is the dimerization and autophosphorylation of PERK (Fig. 6A), and the transcriptomic analysis shown above suggests that MK treatment stimulates PERK phosphorylation. To directly evaluate this possibility, we measured the amounts of P-PERK and total PERK in mock-infected and influenza virus-infected A549 cell cultures treated with various MK concentrations. As a control for PERK phosphorylation, the cultures were treated with thapsigargin, a well-known UPR stimulator (60), and a clear increase in the levels of P-PERK was observed in both mock- and virus-infected cells (Fig. 6B, C+). Treatment of normal cells with MK progressively induced PERK phosphorylation (Fig. 6B, MOCK), while virus infection reduced the P-PERK levels (Fig. 6B, compare FLU with MOCK, untreated). Treatment of virus-infected cells with MK led to the restoration of P-PERK levels to those found in normal cells (Fig. 6B, compare lanes MOCK 0 and FLU 40). In agreement with the results shown in Fig. 2C, the NP accumulation was strongly reduced upon treatment with MK at 40 µM and, interestingly, it was also reduced upon treatment with thapsigargin (Fig. 6, NP, C+). The quantitation of several experiments similar to that presented in Fig. 6B is presented in Fig. 6C and indicates an opposite and statistically significant variation of PERK phosphorylation by virus infection and by MK treatment.

Virus infection is among the various stimuli that induce the UPR, probably because of the rapid demand of protein synthesis and folding capacity made by the virus. Some viruses, like hepatitis C virus and many other positive-polarity RNA viruses, have evolved not only to resist the UPR but also to take advantage of it (reviewed in references 61 and 62), much in the same way that influenza viruses have usurped other signaling pathways to optimize the replication cycle (see below). However, firing of the PERK-mediated UPR leads to protein synthesis attenuation by phosphorylation of the eIF2α translation factor and hence constitutes a strong limitation of the rapid demand exerted by acute influenza virus infection. To test whether other fast-replication viruses are inhibited by MK, we carried out experiments similar to that presented in Fig. 4 with the New Caledonia (H1N1) strain of influenza virus and vesicular stomatitis virus (VSV). Results showed that the protein synthesis of both viruses was strongly reduced under conditions that did not alter cellular protein synthesis (see Fig. S5 in the supplemental material). This observation is in agreement with protein translation being the crucial step in MK-induced virus inhibition, as VSV is a cytoplasmic negative-stranded RNA virus with no nuclear phase.

It is no surprise that, acting as an inducer of PERK phosphorylation (Fig. 6), MK showed up as an inhibitor in the screening based on virus RNP-based GFP expression (Fig. 1), led to preferential reduction of viral protein synthesis, and inhibited virus multiplication in cultured cells (Fig. 2 and 4). Consistent with these findings, treatment of influenza virus-infected cells with GSK-2656157, a specific inhibitor of PERK phosphorylation, led to a dose-dependent increase in viral protein synthesis (Fig. 4C and D). Although the rather low CC_50_-to-IC_50_ ratio observed (Fig. 3 and 5) precluded any use of MK as an influenza virus inhibitor, the results presented support the use of PERK phosphorylation as a target to limit virus multiplication. Being a cellular target, it would have a low probability of eliciting the appearance of resistant virus strains and could be considered complementary to those virus targets already shown to be effective or in the process of development. Conversely, alterations of the PERK-dependent UPR might lead to potential deregulation of other stress response pathways that may have side effects on the host cell. However, since influenza virus infection produces an acute disease that would require only short-term treatment, the consequences of these potential side effects might be less relevant. Although inhibition of other cell signaling pathways has been extensively considered for anti-influenza virus treatment (reviewed in reference 31), the PERK-driven UPR has not been described yet as a potential influenza virus target. However, inhibitors of eIF2α dephosphorylation have been shown to downregulate virus multiplication. Thus, the small molecule salubrinal has been reported to inhibit several PERK/eIF2αphosphatases (63) and be effective against HSV1 (64) and dengue virus infections (65; reviewed in reference 66). Moreover, the inhibitor guanabenz (Wytensin), a drug used for a long time to treat hypertension, was shown to inhibit the GADD34/PPP1R15α-dependent PERK/eIF2α phosphatase specifically (67). Consistent with such action, we show here that treatment of influenza virus-infected cells with guanabenz results in partial inhibition of viral protein synthesis under conditions that do not affect general cellular protein synthesis (Fig. 4E and F).

### The PERK-induced UPR as a potential new anti-influenza virus target.

Influenza virus infection leads to the activation of a number of cell signaling pathways, including those dependent on NF-κB, Raf/MEK/ERK, and phosphatidylinositol 3-kinase (PI3K)/Akt (reviewed in references 30 and 68 to 70). Thus, the expression of some viral proteins leads to the phosphorylation of IκB and the subsequent activation of NF-κB (71), which participates in the cellular innate and inflammatory responses. However, inhibition of this pathway has been shown to interfere with virus replication (72), probably because of a defect in caspase-3 activity and the subsequent retention of progeny RNPs in the nucleus (reviewed in reference 68). Similarly, activation of the Raf/MEK/ERK pathway appears to be important for late events in the virus replication cycle, since its inhibition also leads to the accumulation of progeny RNPs in the nucleus. In this case, an ERK-dependent phosphorylation event in the viral NP or cellular factors seems to fire RNP export as a result of the late accumulation of viral hemagglutinin at the cell surface (29, 73). On the other hand, activation of the PI3K/Akt pathway by virus infection is bimodal. While an early burst of PI3K activation is induced by virus binding and is important for virus entry (74), late stimulation was also observed, which is relevant to avoid the premature appearance of apoptosis and is induced by the viral NS1 protein (26, 27, 75, 76). Thus, the virus makes use of cellular pathways that normally participate in the cellular innate and inflammatory responses to stimulate specific events in its replication cycle.

Contrary to these findings, here we show that influenza virus infection leads to attenuation of the PERK-mediated UPR (Fig. 5A to C) but does not induce general downregulation of the ATF6 or IRE1 arm of this stress response (see Table S1 in the supplemental material). This is in contrast to previous studies showing the stimulation of ATF6- and IRE1-dependent UPR in virus-infected cells (77, 78). However, those investigators analyzed UPR activation at very late times after infection (12 to 48 h), long after the virus infection cycle is finished (around 8 h). Here, we show clear downregulation of PERK phosphorylation as early as 6 h after virus infection (Fig. 6B and C). This observation is in line with the activation in influenza virus-infected cells of p58^IPK^, a cellular inhibitor of PKR that also inhibits PERK autophosphorylation (79), although our data indicate a small transcriptional downregulation upon influenza virus infection (see Table S1). In addition, upregulation of the transcription of GADD34/PPP1R15α, a coactivator of PERK/eIF2α phosphatase, was observed in virus-infected cells (see Table S3), which could also account for the reduced levels of PERK phosphorylation (Fig. 6A to C).

Interestingly, these findings were derived from data obtained after the treatment of infected cells with MK, a known drug that has long been used for the treatment of asthma in humans (reviewed in reference 80). When used at higher doses in influenza virus-infected human cells, MK preferentially reduced viral protein synthesis and accumulation (Fig. 2 and 4) without altering virus transcription or splicing (Fig. 3; see Fig. S3 in the supplemental material). These findings are consistent with the stimulation of PERK phosphorylation observed upon MK treatment of mock-infected cells and the reversal of the virus-induced downregulation of PERK phosphorylation (Fig. 6B and C). Consistently, transcription of GADD34/PPP1R15α was not enhanced in virus-infected cells by treatment with MK (see Table S3 in the supplemental material).

The mechanism by which MK elicits activation of the PERK-mediated UPR but not the ATF6- and IRE1-dependent arms is not clear at this time. Treatment with MK does not downregulate the GADD34/PPP1R15α regulatory cofactor, but it is able to counteract virus-induced GADD34/PPP1R15α overexpression (see Table S3). Anyway, the use of MK in influenza virus-infected cells has uncovered the PERK-mediated UPR as a potential target for anti-influenza virus treatment, which would complement the various virus targets presently under development. In addition, being a cellular target, it may eventually be useful for inhibiting other fast-replicating RNA viruses.

## MATERIALS AND METHODS

### Biological materials.

The HEK 293T cell line (81) was obtained from J. C. de la Torre, the A549 human cell line (82) was obtained from J. A. Melero, and the Huh7 cell line (83) was obtained from P. Gastaminza. The MDCK cell line was purchased from the ATCC. Cell culture was carried out as previously described (84). Influenza virus strains A/Wisconsin/33 (H1N1) (WSN) and A/New Caledonia/20/99 (H1N1) and a recombinant virus containing the M, HA, and NA segments of WSN in the background of A/Victoria/3/75 (H3N2) (VIC) (85) were used in these experiments. Titrations were done in MDCK cells as previously described (86). VSV was provided by R. Alfonso and titrated in BHK21 cells. Plasmids pCMVPB1, pCMVPB2, pCMVPA, and pCMVNP, expressing the influenza virus polymerase subunits and the NP, have been previously described (85). Plasmid pHHclone23, a genomic plasmid expressing a deleted NS RNA segment (clone 23) (57) in negative polarity, was provided by R. Coloma. Antiserum specific for NP was generated by immunization of rabbits with recombinant NP (57). A polyclonal antibody specific for P-PERK (SC-32577) was purchased from Santa Cruz, and a monoclonal antibody specific for actin (ab8226) was purchased from Abcam. The secondary antibodies used for Western blotting were purchased from Sigma.

### Plasmid construction.

To construct plasmid pHHGFP, a PCR fragment containing the gene for enhanced GFP flanked by the conserved sequences of the NS segment was amplified by using as primers 5′ TCAATCA*CGTCTC*TTATTAGTAGAAACAAGGGTGTTTT 3′ and 5′ CAGTATCA*CGTCTC*TGGGAGCAAAAGCAGGGTGACAAAG 3′, which contain the NS-specific sequences (underlined) and BsmBI restriction sites (italics). The PCR fragment was digested with BsmBI and ligated with BsmBI-digested plasmid pHH21 (87).

### Chemical compounds.

The chemical libraries containing 727 compounds that have been clinically tested for a wide variety of indications were obtained from the National Institutes of Health Clinical Collection (NCC-104, NCC-201). MK sodium and T-705 (favipiravir) were obtained from AKOS GmbH (AKOS015833416 and AKOS005166863, respectively), and GSK-2656157 was obtained from MedKoo (406230). Wytensin (guanabenz) was obtained from Sigma (G110). All compounds were dissolved in DMSO at a 10 mM final concentration.

### Library screening.

Cultures of HEK293T cells in 96-well plates were transfected with a mixture of plasmids expressing the polymerase subunits (pCMVPB1, 3 ng; pCMVPB2His, 1.5 ng; pCMVPA, 2.5 ng) and NP (pCMVNP, 120 ng) and a genomic plasmid expressing a viral RNA-like GFP-encoding gene (pHHEGFP, 120 ng) by the calcium phosphate technique (88). As a control, HEK293T cells were transfected with 12 ng of pCAGGsGFP. At 2 hpi, compound stock solutions were diluted to 50 µM in 100 µl of growth medium (DMEM–5% fetal bovine serum [FBS]) and this mixture was added to each well. The cells were incubated at 37°C for 5 days, after which images of each well were collected on a Leica DMI 6000B with an ORCA-R2 digital camera (Hamamatsu). Images were acquired with a 10× (0.30 numerical aperture) objective and a resolution of 1,344 by 1,024 pixels. Quantification of the fluorescent signal was performed with the ImageJ software. Compound toxicity was determined by evaluating the metabolic activity of the cell biomass by the MTT cell viability assay (89).

### Infection assays.

Cells were infected with viruses at the multiplicity of infection (MOI) specified for each experiment. After 1 h, cells were washed with phosphate-buffered saline (PBS) and overlaid with growth medium (DMEM–5% FBS) including the corresponding amount of MK, thapsigargin, GSK-2656157, guanabenz, or DMSO. Supernatants were harvested and used for virus titration by plaque assay in MDCK cells, or cell extracts were prepared for protein analysis.

### Protein analyses.

Protein samples were separated by SDS-polyacrylamide gel electrophoresis and transferred to Immobilon filters. Western blotting was carried out essentially as previously described (90). The membranes were saturated with 3% bovine serum albumin for 1 h and then incubated with the primary antibodies for 1 h at room temperature. For Western blotting of phosphorylated proteins, the membranes were saturated with 5% milk for 1 h and then incubated with the primary antibodies overnight at 4°C. The filters were washed with PBS containing 0.25% Tween 20 (Tris-buffered saline containing 0.25% Tween 20 for phosphorylated proteins) and incubated with the appropriate secondary antibody conjugated to horseradish peroxidase. After further washing as described above, the filters were developed by enhanced chemiluminescence. The procedures for protein labeling *in vivo* have been previously described (91). Cultures were washed, incubated for 1 h in a DMEM medium lacking methionine-cysteine, and labeled with [^35^S]methionine-cysteine to a final concentration of 50 µCi/ml. After incubation for 1 h, total extracts were prepared in Laemmli sample buffer and processed by polyacrylamide gel electrophoresis and autoradiography. For quantification of cell protein synthesis, the complete lanes were used, while quantification of viral protein synthesis was carried out with the specific protein bands indicated in the figures.

### Transcription activity of viral RNPs.

Recombinant RNPs containing the clone 23 genomic RNAs were generated and amplified in 8 × 10^6^ HEK293T cells by transfection of a mixture of plasmids expressing the polymerase subunits (pCMVPB1, 3 µg; pCMVPB2His, 3 µg; pCMVPA, 600 ng) and NP (pCMVNP, 12 µg) and a genomic plasmid (pHHclone23, 12 µg) by the calcium phosphate technique (88). At 24 h posttransfection, cell extracts were prepared and the enzymatic activity of viral RNPs was determined *in vitro* with β-globin mRNA as the primer donor as previously described (57).

### RT-qPCR.

Quantification of primary transcription was carried out by reverse transcription-quantitative PCR (RT-qPCR) as described previously (92). A 5.5-µl mixture containing 200 ng of total RNA and 10 pmol of a tagged primer specific for NP mRNA (5′ CCAGATCGTTCGAGTCGTTTTTTTTTTTTTTTTTAATTGTCGTAC 3′) were heated for 10 min at 65°C, chilled immediately on ice for 5 min, and then heated again at 60°C. After 5 min, 14.5 µl of a preheated reaction mixture containing 4 µl of First-Strand Buffer (Invitrogen), 1 µl of 0.1 M dithiothreitol, 1 µl of a mixture of deoxynucleoside triphosphates (each at 10 mM), 1 µl of Superscript II reverse transcriptase (50 U/µl; Invitrogen), 1 µl of RNasin Plus RNase inhibitor (40 U/μl; Promega), and 6.5 µl of saturated trehalose was added and the mixture was incubated for 1 h. Real-time qPCR was performed with Power SYBR green PCR master mix (Applied Biosystems). Four microliters of a 10-fold dilution of the cDNA was added to a qPCR mixture containing 10 µl of SYBR green qPCR SuperMix, 1.5 µl of forward primer (10 µM), 1.5 µl of reverse primer (10 µM), and 3 µl of double-distilled water. The sequences of the primers specific for the NP mRNA are 5′ CCAGATCGTTCGAGTCGT 3′ and 5′ GTCGTGCCCTCTTTTGACAT 3′. The PCR was carried out in a PRISM 7000 sequence detection system (Applied Biosystems) with 1 cycle of 50°C for 2 min, followed by 1 cycle of 95°C for 10 min and 40 cycles of 95°C for 15 s and 60°C for 1 min. The cycle threshold was determined with the SDS analytical software (Applied Biosystems).

### RNA purification, cDNA synthesis, and deep sequencing.

For RNA extraction, cell pellets containing 2.5 × 10^6^ cells were resuspended in 1 ml of Trizol reagent (Invitrogen) and the RNA was purified as recommended by the manufacturer. The RNA was digested with RNase-free DNase (1 U/mg) for 1 h at 37°C, extracted with phenol-chloroform-isoamyl alcohol, and precipitated with ethanol. The purified RNA was resuspended in nuclease-free water, and the absorbance at 260 nm was measured (NanoDrop ND-1000). For deep sequencing, rRNA was removed with the Ribo-Zero rRNA removal kit (catalog no. RZH1046; Illumina). Each RNA preparation was monitored with an Agilent 2100 Bioanalyzer (Agilent Technologies). cDNA was synthesized with reverse transcriptase (SuperScript II, catalog no. 18064-014; Invitrogen) and random primers. The second strand of the cDNA incorporated dUTP instead of dTTP. Double-stranded DNA was subjected to A tailing and ligation of the bar-coded TruSeq adapters. Purification steps were done with AMPure XP beads. Library amplification was performed by PCR with the primer cocktail supplied in the kit. Final libraries were analyzed with Agilent DNA 1000 chips to estimate the quantity and check size distribution and were then quantified by qPCR with the KAPA Library Quantification kit (catalog no. KK4835; KAPA Biosystems) prior to amplification with Illumina cBot. Sequencing was done with Illumina HiSeq by using single reads of 50 nt.

### Transcriptome sequencing (RNA-Seq) data analysis.

The quality of Illumina reads (50 nt) was checked with FastQC (http://www.bioinformatics.babraham.ac.uk/projects/fastqc/). No additional read filtering was needed. TopHat2 (93) was used to align all reads against a human (GRCh38) or influenza virus (A/Victoria/3/75 [H3N2]) genome. The htseq-count function of the HTSeq package (94) was used to assign read counts with all of the human genes (GRCh38 Ensemble release 76). The DESeq2 package (95) of the bioconductor project (http://www.bioconductor.org) was used to determine the statistical significance of the differential expression of genes, and the results are presented as adjusted *P* values (96) based on two biological replicates per sample. Detection of outliers based on Cook’s distance was disabled (cooksCutoff = FALSE). FIESTA viewer (97) was used for real-time graphic evaluation of the application of different combinations of fold changes and FDR thresholds for filtering of gene expression results.

### Nucleotide sequence accession number.

The RNA-Seq data obtained in this study have been deposited in the Gene Expression Omnibus database under accession no. GSE68673.

## SUPPLEMENTAL MATERIAL

Figure S1  Screening parameters. The average values determined for 96 GFP-positive wells (μ_pos_), negative wells (μ_neg_), and the corresponding standard deviations (δ_pos_ and δ_neg_) are shown. These values were used to calculate the reproducibility (Z), percent coefficient of variation (%CV), signal-to-background (S/B) ratio, and signal-to-noise (S/N) ratio with the formulas shown. Download Figure S1, PDF file, 0.1 MB

Figure S2  Characterization of the MK preparation used for screening. (A) Chemical structure of MK obtained from the PubChem database (CID 23663996) corresponding to the formula C_35_H_35_ClNNaO_3_S and a molecular mass of 608.16 g/mol. The mass spectra of samples of the compound MK obtained from the NIH library (B) and AKOS GmbH (C), as determined by matrix-assisted laser desorption ionization--time of flight tandem mass spectrometry, are shown. Peaks correspond to ionized fragments of the compound generated by laser irradiation and separated by mass/charge ratios with a mass spectrometer. Download Figure S2, PDF file, 0.5 MB

Figure S3  Splicing of influenza virus mRNA is not altered by MK treatment. Cultures of human A549 cells were infected with influenza virus at an MOI of 3 PFU/cell and then treated with 20 µM MK or the corresponding amount of DMSO. Total cell RNA was isolated at 6 hpi, and rRNA was removed. Viral RNA of each segment was determined by deep sequencing and classified according to the virus segment and polarity. The splicing efficiencies of segments 7 (M2/total M) and 8 (NEP/total NS) are presented as reads in splice junctions versus the total reads in each segment mRNA. Download Figure S3, PDF file, 0.2 MB

Figure S4  Functional protein association network of the genes differentially expressed in influenza virus-infected cells upon treatment with MK. Shown is the protein-protein interaction network as visualized by the STRING database. The nodes represent proteins, red circles contain most of the proteins differentially expressed in influenza virus-infected cells upon treatment with MK, and the lines represent the predicted functional associations. The associations were inferred from several types of evidence from the STRING database: the presence of experimental evidence (pink line), text mining evidence from abstracts of the scientific literature (yellow line), information from databases (light blue), sequence homology (light gray), co-occurrence evidence (dark blue), and coexpression evidence (dark gray). Retrieved from STRING 9.1, 2015. Download Figure S4, PDF file, 0.2 MB

Figure S5  MK inhibits viral protein synthesis. Cultures of human A549 cells were infected with the New Caledonia (H1N1) or VIC (H3N2) strain of influenza virus or with VSV at an MOI of 3 PFU/cell and then treated with 40 µM MK or the corresponding amount of DMSO. At 6 hpi, the cultures were pulse-labeled with [^35^S]methionine-cysteine and total protein extracts were prepared. (A) The samples were analyzed by polyacrylamide gel electrophoresis and autoradiography. The mobility of some of the virus-specific proteins (stars for influenza virus and arrows for VSV) is indicated to the right. (B) Quantification of the signals of DMSO-treated (blue) and MK-treated (red) samples. Download Figure S5, PDF file, 0.9 MB

Table S1  Alterations of the ATF6- and IRE1-dependent unfolded protein response pathways by influenza virus infection (FLU) and/or MK treatment. Shown are the genes regulated by the transcription factors ATF6 and IRE1. The identification (ID) code in the Ensembl database is specified, as well as the FC and the FDR. Table S1, PDF file, 0.1 MB

Table S2  Lack of alteration of genes downregulated by virus infection upon MK treatment. Shown are the genes downregulated by influenza virus infection (FLU versus MOCK; cutoff values, FC of less than −3 and FDR of <10^−3^) and the lack of their modification by treatment with MK (FLU-MK versus FLU, MOCK-MK versus MOCK). The identification (ID) code in the Ensembl database is specified, as well as the FC and the FDR. Table S2, PDF file, 0.1 MB

Table S3  Alterations of GADD34/PPP1R15α transcription by influenza virus infection (FLU) and/or MK treatment. The identification (ID) code in the Ensembl database is specified, as well as the FC and the FDR. Table S3, PDF file, 0.1 MB

## References

[1] Molinari NA, Ortega-Sanchez IR, Messonnier ML, Thompson WW, Wortley PM, Weintraub E, Bridges CB 2007 The annual impact of seasonal influenza in the US: measuring disease burden and costs. Vaccine 25:5086–5096. doi:10.1016/j.vaccine.2007.03.046. 17544181

[2] Taubenberger JK, Morens DM 2006 1918 influenza: the mother of all pandemics. Emerg Infect Dis 12:15–22. doi:10.3201/eid1201.050979. 16494711 PMC3291398

[3] Shaw M, Palese P 2013 Orthomyxoviridae, p 1151–1185. *In* Knipe DM, Howley P (ed), Fields virology, vol 1, 6th ed. Lippincott Williams & Wilkins, Philadelphia, PA.

[4] Pons MW 1973 The inhibition of influenza virus RNA synthesis by actinomycin D and cycloheximide. Virology 51:120–128. doi:10.1016/0042-6822(73)90372-3. 4734322

[5] Huang TS, Palese P, Krystal M 1990 Determination of influenza virus proteins required for genome replication. J Virol 64:5669–5673. 2214032 10.1128/jvi.64.11.5669-5673.1990 PMC248627

[6] Eisfeld AJ, Neumann G, Kawaoka Y 2015 At the centre: influenza A virus ribonucleoproteins. Nat Rev Microbiol 13:28–41. doi:10.1038/nrmicro3367. 25417656 PMC5619696

[7] Fodor E 2013 The RNA polymerase of influenza A virus: mechanisms of viral transcription and replication. Acta Virol 57:113–122. doi:10.4149/av_2013_02_113. 23600869

[8] Martín-Benito J, Ortín J 2013 Influenza virus transcription and replication. Adv Virus Res 87:113–137. doi:10.1016/B978-0-12-407698-3.00004-1. 23809922

[9] Resa-Infante P, Jorba N, Coloma R, Ortín J 2011 The influenza virus RNA synthesis machine: advances in its structure and function. RNA Biol 8:207–215. doi:10.4161/rna.8.1.15302. 21358279 PMC3127100

[10] Rossman JS, Lamb RA 2011 Influenza virus assembly and budding. Virology 411:229–236. doi:10.1016/j.virol.2010.12.003. 21237476 PMC3086653

[11] De Clercq E 2006 Antiviral agents active against influenza A viruses. Nat Rev Drug Discov 5:1015–1025. doi:10.1038/nrd2175. 17139286 PMC7097821

[12] Furuta Y, Gowen BB, Takahashi K, Shiraki K, Smee DF, Barnard DL 2013 Favipiravir (T-705), a novel viral RNA polymerase inhibitor. Antiviral Res 100:446–454. doi:10.1016/j.antiviral.2013.09.015. 24084488 PMC3880838

[13] Byrn RA, Jones SM, Bennett HB, Bral C, Clark MP, Jacobs MD, Kwong AD, Ledeboer MW, Leeman JR, McNeil CF, Murcko MA, Nezami A, Perola E, Rijnbrand R, Saxena K, Tsai AW, Zhou Y, Charifson PS 2015 Preclinical activity of VX-787, a first in class, orally bioavailable inhibitor of the influenza virus polymerase PB2 subunit. Antimicrob Agents Chemother 59:1569–1582. doi:10.1128/AAC.04623-14. 25547360 PMC4325764

[14] Clark MP, Ledeboer MW, Davies I, Byrn RA, Jones SM, Perola E, Tsai A, Jacobs M, Nti-Addae K, Bandarage UK, Boyd MJ, Bethiel RS, Court JJ, Deng H, Duffy JP, Dorsch WA, Farmer LJ, Gao H, Gu W, Jackson K, Jacobs DH, Kennedy JM, Ledford B, Liang J, Maltais F, Murcko M, Wang T, Wannamaker MW, Bennett HB, Leeman JR, McNeil C, Taylor WP, Memmott C, Jiang M, Rijnbrand R, Bral C, Germann U, Nezami A, Zhang Y, Salituro FG, Bennani YL, Charifson PS 2014 Discovery of a novel, first-in-class, orally bioavailable azaindole inhibitor (VX-787) of influenza PB2. J Med Chem 57:6668–6678. doi:10.1021/jm5007275. 25019388

[15] DuBois RM, Slavish PJ, Baughman BM, Yun MK, Bao J, Webby RJ, Webb TR, White SW 2012 Structural and biochemical basis for development of influenza virus inhibitors targeting the PA endonuclease. PLoS Pathog 8:e1002830. doi:10.1371/journal.ppat.1002830. 22876176 PMC3410894

[16] Gerritz SW, Cianci C, Kim S, Pearce BC, Deminie C, Discotto L, McAuliffe B, Minassian BF, Shi S, Zhu S, Zhai W, Pendri A, Li G, Poss MA, Edavettal S, McDonnell PA, Lewis HA, Maskos K, Mörtl M, Kiefersauer R, Steinbacher S, Baldwin ET, Metzler W, Bryson J, Healy MD, Philip T, Zoeckler M, Schartman R, Sinz M, Leyva-Grado VH, Hoffmann HH, Langley DR, Meanwell NA, Krystal M 2011 Inhibition of influenza virus replication via small molecules that induce the formation of higher-order nucleoprotein oligomers. Proc Natl Acad Sci U S A 108:15366–15371. doi:10.1073/pnas.1107906108. 21896751 PMC3174639

[17] Kowalinski E, Zubieta C, Wolkerstorfer A, Szolar OH, Ruigrok RW, Cusack S 2012 Structural analysis of specific metal chelating inhibitor binding to the endonuclease domain of influenza pH1N1 (2009) polymerase. PLoS Pathog 8:e1002831. doi:10.1371/journal.ppat.1002831. 22876177 PMC3410856

[18] Lejal N, Tarus B, Bouguyon E, Chenavas S, Bertho N, Delmas B, Ruigrok RW, Di Primo C, Slama-Schwok A 2013 Structure-based discovery of the novel antiviral properties of naproxen against the nucleoprotein of influenza A virus. Antimicrob Agents Chemother 57:2231–2242. doi:10.1128/AAC.02335-12. 23459490 PMC3632891

[19] Ortigoza MB, Dibben O, Maamary J, Martinez-Gil L, Leyva-Grado VH, Abreu P Jr., Ayllon J, Palese P, Shaw ML 2012 A novel small molecule inhibitor of influenza A viruses that targets polymerase function and indirectly induces interferon. PLoS Pathog 8:e1002668. doi:10.1371/journal.ppat.1002668. 22577360 PMC3343121

[20] Stevaert A, Dallocchio R, Dessì A, Pala N, Rogolino D, Sechi M, Naesens L 2013 Mutational analysis of the binding pockets of the diketo acid inhibitor L-742,001 in the influenza virus PA endonuclease. J Virol 87:10524–10538. doi:10.1128/JVI.00832-13. 23824822 PMC3807387

[21] Su CY, Cheng TJ, Lin MI, Wang SY, Huang WI, Lin-Chu SY, Chen YH, Wu CY, Lai MM, Cheng WC, Wu YT, Tsai MD, Cheng YS, Wong CH 2010 High-throughput identification of compounds targeting influenza RNA-dependent RNA polymerase activity. Proc Natl Acad Sci U S A 107:19151–19156. doi:10.1073/pnas.1013592107. 20974907 PMC2984200

[22] Vanderlinden E, Naesens L 2014 Emerging antiviral strategies to interfere with influenza virus entry. Med Res Rev 34:301–339. doi:10.1002/med.21289. 23801557 PMC7168512

[23] Kiso M, Mitamura K, Sakai-Tagawa Y, Shiraishi K, Kawakami C, Kimura K, Hayden FG, Sugaya N, Kawaoka Y 2004 Resistant influenza A viruses in children treated with oseltamivir: descriptive study. Lancet 364:759–765. doi:10.1016/S0140-6736(04)16934-1. 15337401

[24] Dharan NJ, Gubareva LV, Meyer JJ, Okomo-Adhiambo M, McClinton RC, Marshall SA, St George K, Epperson S, Brammer L, Klimov AI, Bresee JS, Fry AM 2009 Infections with oseltamivir-resistant influenza A(H1N1) virus in the United States. JAMA 301:1034–1041. doi:10.1001/jama.2009.294. 19255110

[25] Ehrhardt C, Rückle A, Hrincius ER, Haasbach E, Anhlan D, Ahmann K, Banning C, Reiling SJ, Kühn J, Strobl S, Vitt D, Leban J, Planz O, Ludwig S 2013 The NF-kappaB inhibitor SC75741 efficiently blocks influenza virus propagation and confers a high barrier for development of viral resistance. Cell Microbiol 15:1198–1211. doi:10.1111/cmi.12108. 23320394

[26] Ehrhardt C, Wolff T, Pleschka S, Planz O, Beermann W, Bode JG, Schmolke M, Ludwig S 2007 Influenza A virus NS1 protein activates the PI3K/Akt pathway to mediate antiapoptotic signaling responses. J Virol 81:3058–3067. doi:10.1128/JVI.02082-06. 17229704 PMC1866065

[27] Hale BG, Jackson D, Chen YH, Lamb RA, Randall RE 2006 Influenza A virus NS1 protein binds p85beta and activates phosphatidylinositol-3-kinase signaling. Proc Natl Acad Sci U S A 103:14194–14199. doi:10.1073/pnas.0606109103. 16963558 PMC1599933

[28] Ludwig S, Wolff T, Ehrhardt C, Wurzer WJ, Reinhardt J, Planz O, Pleschka S 2004 MEK inhibition impairs influenza B virus propagation without emergence of resistant variants. FEBS Lett 561:37–43. doi:10.1016/S0014-5793(04)00108-5. 15013748

[29] Pleschka S, Wolff T, Ehrhardt C, Hobom G, Planz O, Rapp UR, Ludwig S 2001 Influenza virus propagation is impaired by inhibition of the Raf/MEK/ERK signalling cascade. Nat Cell Biol 3:301–305. doi:10.1038/35060098. 11231581

[30] Hale BG, Randall RE 2007 PI3K signalling during influenza A virus infections. Biochem Soc Trans 35:186–187. doi:10.1042/BST0350186. 17371234

[31] Planz O 2013 Development of cellular signaling pathway inhibitors as new antivirals against influenza. Antiviral Res 98:457–468. doi:10.1016/j.antiviral.2013.04.008. 23603495

[32] Falcón AM, Fortes P, Marión RM, Beloso A, Ortín J 1999 Interaction of influenza virus NS1 protein and the human homologue of Staufen in vivo and in vitro. Nucleic Acids Res 27:2241–2247. doi:10.1093/nar/27.11.2241. 10325410 PMC148787

[33] Nemeroff ME, Barabino SM, Li Y, Keller W, Krug RM 1998 Influenza virus NS1 protein interacts with the cellular 30-kDa subunit of CPSF and inhibits 3′ end formation of cellular pre-mRNAs. Mol Cell 1:991–1000. doi:10.1016/S1097-2765(00)80099-4. 9651582

[34] O’Neill RE, Palese P 1995 NPI-1, the human homolog of SRP-1, interacts with influenza virus nucleoprotein. Virology 206:116–125. doi:10.1016/S0042-6822(95)80026-3. 7831767

[35] Shapira SD, Gat-Viks I, Shum BO, Dricot A, de Grace MM, Wu L, Gupta PB, Hao T, Silver SJ, Root DE, Hill DE, Regev A, Hacohen N 2009 A physical and regulatory map of host-influenza interactions reveals pathways in H1N1 infection. Cell 139:1255–1267. doi:10.1016/j.cell.2009.12.018. 20064372 PMC2892837

[36] Jorba N, Juarez S, Torreira E, Gastaminza P, Zamarreño N, Albar JP, Ortín J 2008 Analysis of the interaction of influenza virus polymerase complex with human cell factors. Proteomics 8:2077–2088. doi:10.1002/pmic.200700508. 18491320

[37] Mayer D, Molawi K, Martínez-Sobrido L, Ghanem A, Thomas S, Baginsky S, Grossmann J, García-Sastre A, Schwemmle M 2007 Identification of cellular interaction partners of the influenza virus ribonucleoprotein complex and polymerase complex using proteomic-based approaches. J Proteome Res 6:672–682. doi:10.1021/pr060432u. 17269724 PMC2577182

[38] Hutchinson EC, Charles PD, Hester SS, Thomas B, Trudgian D, Martinez-Alonso M, Fodor E 2014 Conserved and host-specific features of influenza virion architecture. Nat Commun 5:4816. doi:10.1038/ncomms5816. 25226414 PMC4167602

[39] Shaw ML, Stone KL, Colangelo CM, Gulcicek EE, Palese P 2008 Cellular proteins in influenza virus particles. PLoS Pathog 4:e1000085. doi:10.1371/journal.ppat.1000085. 18535660 PMC2390764

[40] Brass AL, Huang IC, Benita Y, John SP, Krishnan MN, Feeley EM, Ryan BJ, Weyer JL, van der Weyden L, Fikrig E, Adams DJ, Xavier RJ, Farzan M, Elledge SJ 2009 The IFITM proteins mediate cellular resistance to influenza A H1N1 virus, west Nile virus, and dengue virus. Cell 139:1243–1254. doi:10.1016/j.cell.2009.12.017. 20064371 PMC2824905

[41] Hao L, Sakurai A, Watanabe T, Sorensen E, Nidom CA, Newton MA, Ahlquist P, Kawaoka Y 2008 Drosophila RNAi screen identifies host genes important for influenza virus replication. Nature 454:890–893. doi:10.1038/nature07151. 18615016 PMC2574945

[42] Karlas A, Machuy N, Shin Y, Pleissner KP, Artarini A, Heuer D, Becker D, Khalil H, Ogilvie LA, Hess S, Mäurer AP, Müller E, Wolff T, Rudel T, Meyer TF 2010 Genome-wide RNAi screen identifies human host factors crucial for influenza virus replication. Nature 463:818–822. doi:10.1038/nature08760. 20081832

[43] König R, Stertz S, Zhou Y, Inoue A, Hoffmann HH, Bhattacharyya S, Alamares JG, Tscherne DM, Ortigoza MB, Liang Y, Gao Q, Andrews SE, Bandyopadhyay S, De Jesus P, Tu BP, Pache L, Shih C, Orth A, Bonamy G, Miraglia L, Ideker T, Garcia-Sastre A, Young JA, Palese P, Shaw ML, Chanda SK 2010 Human host factors required for influenza virus replication. Nature 463:813–817. doi:10.1038/nature08699. 20027183 PMC2862546

[44] Stertz S, Shaw ML 2011 Uncovering the global host cell requirements for influenza virus replication via RNAi screening. Microbes Infect 13:516–525. doi:10.1016/j.micinf.2011.01.012. 21276872 PMC3071880

[45] Fornek JL, Gillim-Ross L, Santos C, Carter V, Ward JM, Cheng LI, Proll S, Katze MG, Subbarao K 2009 A single-amino-acid substitution in a polymerase protein of an H5N1 influenza virus is associated with systemic infection and impaired T-cell activation in mice. J Virol 83:11102–11115. doi:10.1128/JVI.00994-09. 19692471 PMC2772766

[46] Geiss GK, An MC, Bumgarner RE, Hammersmark E, Cunningham D, Katze MG 2001 Global impact of influenza virus on cellular pathways is mediated by both replication-dependent and -independent events. J Virol 75:4321–4331. doi:10.1128/JVI.75.9.4321-4331.2001. 11287581 PMC114177

[47] Kash JC, Basler CF, García-Sastre A, Carter V, Billharz R, Swayne DE, Przygodzki RM, Taubenberger JK, Katze MG, Tumpey TM 2004 Global host immune response: pathogenesis and transcriptional profiling of type A influenza viruses expressing the hemagglutinin and neuraminidase genes from the 1918 pandemic virus. J Virol 78:9499–9511. doi:10.1128/JVI.78.17.9499-9511.2004. 15308742 PMC506954

[48] Coombs KM, Berard A, Xu W, Krokhin O, Meng X, Cortens JP, Kobasa D, Wilkins J, Brown EG 2010 Quantitative proteomic analyses of influenza virus-infected cultured human lung cells. J Virol 84:10888–10906. doi:10.1128/JVI.00431-10. 20702633 PMC2950599

[49] Vester D, Rapp E, Gade D, Genzel Y, Reichl U 2009 Quantitative analysis of cellular proteome alterations in human influenza A virus-infected mammalian cell lines. Proteomics 9:3316–3327. doi:10.1002/pmic.200800893. 19504497

[50] de Chassey B, Meyniel-Schicklin L, Vonderscher J, André P, Lotteau V 2014 Virus-host interactomics: new insights and opportunities for antiviral drug discovery. Genome Med 6:115. doi:10.1186/s13073-014-0115-1. 25593595 PMC4295275

[51] Kilcher S, Mercer J 2014 Next generation approaches to study virus entry and infection. Curr Opin Virol 4:8–14. doi:10.1016/j.coviro.2013.10.002. 24525289

[52] Müller KH, Kakkola L, Nagaraj AS, Cheltsov AV, Anastasina M, Kainov DE 2012 Emerging cellular targets for influenza antiviral agents. Trends Pharmacol Sci 33:89–99. doi:10.1016/j.tips.2011.10.004. 22196854

[53] Shaw ML 2011 The host interactome of influenza virus presents new potential targets for antiviral drugs. Rev Med Virol 21:358–369. doi:10.1002/rmv.703. 21823192 PMC3207218

[54] Jorba N, Coloma R, Ortín J 2009 Genetic trans-complementation establishes a new model for influenza virus RNA transcription and replication. PLoS Pathog 5:e1000462. doi:10.1371/journal.ppat.1000462. 19478885 PMC2682650

[55] Zhang JH, Chung TD, Oldenburg KR 1999 A simple statistical parameter for use in evaluation and validation of high throughput screening assays. J Biomol Screen 4:67–73. doi:10.1177/108705719900400206. 10838414

[56] Sidwell RW, Bailey KW, Wong MH, Huffman JH 1995 In vitro and in vivo sensitivity of a non-mouse-adapted influenza A (Beijing) virus infection to amantadine and ribavirin. Chemotherapy 41:455–461. doi:10.1159/000239382. 8529436

[57] Coloma R, Valpuesta JM, Arranz R, Carrascosa JL, Ortín J, Martín-Benito J 2009 The structure of a biologically active influenza virus ribonucleoprotein complex. PLoS Pathog 5:e1000491. doi:10.1371/journal.ppat.1000491. 19557158 PMC2695768

[58] Walter P, Ron D 2011 The unfolded protein response: from stress pathway to homeostatic regulation. Science 334:1081–1086. doi:10.1126/science.1209038. 22116877

[59] Kadowaki H, Nishitoh H 2013 Signaling pathways from the endoplasmic reticulum and their roles in disease. Genes (Basel) 4:306–333. doi:10.3390/genes4030306. 24705207 PMC3924831

[60] Wong WL, Brostrom MA, Kuznetsov G, Gmitter-Yellen D, Brostrom CO 1993 Inhibition of protein synthesis and early protein processing by thapsigargin in cultured cells. Biochem J 289:71–79. doi:10.1042/bj2890071. 8424774 PMC1132132

[61] Blázquez AB, Escribano-Romero E, Merino-Ramos T, Saiz JC, Martín-Acebes MA 2014 Stress responses in flavivirus-infected cells: activation of unfolded protein response and autophagy. Front Microbiol 5:266. doi:10.3389/fmicb.2014.00266. 24917859 PMC4042264

[62] Vasallo C, Gastaminza P 2015 Cellular stress responses in hepatitis C virus infection: mastering a two-edged sword. Virus Res 209:100–117. doi:10.1016/j.virusres.2015.03.013. 25836277

[63] Boyce M, Bryant KF, Jousse C, Long K, Harding HP, Scheuner D, Kaufman RJ, Ma D, Coen DM, Ron D, Yuan J 2005 A selective inhibitor of eIF2alpha dephosphorylation protects cells from ER stress. Science 307:935–939. doi:10.1126/science.1101902. 15705855

[64] Bryant KF, Macari ER, Malik N, Boyce M, Yuan J, Coen DM 2008 ICP34.5-dependent and -independent activities of salubrinal in herpes simplex virus-1 infected cells. Virology 379:197–204. doi:10.1016/j.virol.2008.06.028. 18684481 PMC2665023

[65] Umareddy I, Pluquet O, Wang QY, Vasudevan SG, Chevet E, Gu F 2007 Dengue virus serotype infection specifies the activation of the unfolded protein response. Virol J 4:91. doi:10.1186/1743-422X-4-91. 17888185 PMC2045667

[66] Fullwood MJ, Zhou W, Shenolikar S 2012 Targeting phosphorylation of eukaryotic initiation factor-2alpha to treat human disease. Prog Mol Biol Transl Sci 106:75–106. doi:10.1016/B978-0-12-396456-4.00005-5. 22340715

[67] Tsaytler P, Harding HP, Ron D, Bertolotti A 2011 Selective inhibition of a regulatory subunit of protein phosphatase 1 restores proteostasis. Science 332:91–94. doi:10.1126/science.1201396. 21385720

[68] Ludwig S, Planz O 2008 Influenza viruses and the NF-kappaB signaling pathway—towards a novel concept of antiviral therapy. Biol Chem 389:1307–1312. doi:10.1515/BC.2008.148. 18713017

[69] Pleschka S 2008 RNA viruses and the mitogenic Raf/MEK/ERK signal transduction cascade. Biol Chem 389:1273–1282. doi:10.1515/BC.2008.145. 18713014

[70] Ehrhardt C, Ludwig S 2009 A new player in a deadly game: influenza viruses and the PI3K/Akt signalling pathway. Cell Microbiol 11:863–871. doi:10.1111/j.1462-5822.2009.01309.x. 19290913 PMC7162392

[71] Flory E, Kunz M, Scheller C, Jassoy C, Stauber R, Rapp UR, Ludwig S 2000 Influenza virus-induced NF-kappaB-dependent gene expression is mediated by overexpression of viral proteins and involves oxidative radicals and activation of IkappaB kinase. J Biol Chem 275:8307–8314. doi:10.1074/jbc.275.12.8307. 10722660

[72] Wurzer WJ, Ehrhardt C, Pleschka S, Berberich-Siebelt F, Wolff T, Walczak H, Planz O, Ludwig S 2004 NF-kappaB-dependent induction of tumor necrosis factor-related apoptosis-inducing ligand (TRAIL) and Fas/FasL is crucial for efficient influenza virus propagation. J Biol Chem 279:30931–30937. doi:10.1074/jbc.M403258200. 15143063

[73] Marjuki H, Alam MI, Ehrhardt C, Wagner R, Planz O, Klenk HD, Ludwig S, Pleschka S 2006 Membrane accumulation of influenza A virus hemagglutinin triggers nuclear export of the viral genome via protein kinase Calpha-mediated activation of ERK signaling. J Biol Chem 281:16707–16715. doi:10.1074/jbc.M510233200. 16608852

[74] Ehrhardt C, Marjuki H, Wolff T, Nürnberg B, Planz O, Pleschka S, Ludwig S 2006 Bivalent role of the phosphatidylinositol-3-kinase (PI3K) during influenza virus infection and host cell defence. Cell Microbiol 8:1336–1348. doi:10.1111/j.1462-5822.2006.00713.x. 16882036

[75] Shin YK, Liu Q, Tikoo SK, Babiuk LA, Zhou Y 2007 Influenza A virus NS1 protein activates the phosphatidylinositol 3-kinase (PI3K)/Akt pathway by direct interaction with the p85 subunit of PI3K. J Gen Virol 88:13–18. doi:10.1099/vir.0.82419-0. 17170431

[76] Zhirnov OP, Klenk HD 2007 Control of apoptosis in influenza virus-infected cells by up-regulation of Akt and p53 signaling. Apoptosis 12:1419–1432. doi:10.1007/s10495-007-0071-y. 17468837

[77] Hassan IH, Zhang MS, Powers LS, Shao JQ, Baltrusaitis J, Rutkowski DT, Legge K, Monick MM 2012 Influenza A viral replication is blocked by inhibition of the inositol-requiring enzyme 1 (IRE1) stress pathway. J Biol Chem 287:4679–4689. doi:10.1074/jbc.M111.284695. 22194594 PMC3281634

[78] Roberson EC, Tully JE, Guala AS, Reiss JN, Godburn KE, Pociask DA, Alcorn JF, Riches DW, Dienz O, Janssen-Heininger YM, Anathy V 2012 Influenza induces endoplasmic reticulum stress, caspase-12-dependent apoptosis, and c-Jun N-terminal kinase-mediated transforming growth factor-beta release in lung epithelial cells. Am J Respir Cell Mol Biol 46:573–581. doi:10.1165/rcmb.2010-0460OC. 21799120 PMC3359902

[79] Yan W, Frank CL, Korth MJ, Sopher BL, Novoa I, Ron D, Katze MG 2002 Control of PERK eIF2alpha kinase activity by the endoplasmic reticulum stress-induced molecular chaperone P58IPK. Proc Natl Acad Sci U S A 99:15920–15925. doi:10.1073/pnas.252341799. 12446838 PMC138540

[80] Jarvis B, Markham A 2000 Montelukast: a review of its therapeutic potential in persistent asthma. Drugs 59:891–928. doi:10.2165/00003495-200059040-00015. 10804041

[81] DuBridge RB, Tang P, Hsia HC, Leong PM, Miller JH, Calos MP 1987 Analysis of mutation in human cells by using an Epstein-Barr virus shuttle system. Mol Cell Biol 7:379–387. doi:10.1128/MCB.7.1.379. 3031469 PMC365079

[82] Giard DJ, Aaronson SA, Todaro GJ, Arnstein P, Kersey JH, Dosik H, Parks WP 1973 *In vitro* cultivation of human tumors: establishment of cell lines derived from a series of solid tumors. J Natl Cancer Inst 51:1417–1423. 4357758 10.1093/jnci/51.5.1417

[83] Sainz B Jr., Barretto N, Uprichard SL 2009 Hepatitis C virus infection in phenotypically distinct Huh7 cell lines. PLoS One 4:e6561. doi:10.1371/journal.pone.0006561. 19668344 PMC2720605

[84] Ortín J, Nájera R, López C, Dávila M, Domingo E 1980 Genetic variability of Hong Kong (H3N2) influenza viruses: spontaneous mutations and their location in the viral genome. Gene 11:319–331. doi:10.1016/0378-1119(80)90072-4. 6783473

[85] Falcón AM, Marión RM, Zürcher T, Gómez P, Portela A, Nieto A, Ortín J 2004 Defective RNA replication and late gene expression in temperature-sensitive (A/Victoria/3/75) influenza viruses expressing deleted forms of NS1 protein. J Virol 78:3880–3888. doi:10.1128/JVI.78.8.3880-3888.2004. 15047804 PMC374278

[86] Tobita K, Sugiura A, Enomote C, Furuyama M 1975 Plaque-assay and primary isolation of influenza A viruses in an established line of canine kidney cells (MDCK) in the presence of trypsin. Med Microbiol Immunol 162:9–14. doi:10.1007/BF02123572. 1214709

[87] Neumann G, Watanabe T, Ito H, Watanabe S, Goto H, Gao P, Hughes M, Perez DR, Donis R, Hoffmann E, Hobom G, Kawaoka Y 1999 Generation of influenza A viruses entirely from cloned cDNAs. Proc Natl Acad Sci U S A 96:9345–9350. doi:10.1073/pnas.96.16.9345. 10430945 PMC17785

[88] Wigler M, Pellicer A, Silverstein S, Axel R, Urlaub G, Chasin L 1979 DNA-mediated transfer of the adenine phosphoribosyltransferase locus into mammalian cells. Proc Natl Acad Sci U S A 76:1373–1376. doi:10.1073/pnas.76.3.1373. 286319 PMC383253

[89] Levitz SM, Diamond RD 1985 A rapid colorimetric assay of fungal viability with the tetrazolium salt MTT. J Infect Dis 152:938–945. doi:10.1093/infdis/152.5.938. 2413145

[90] Marión RM, Zürcher T, de la Luna S, Ortín J 1997 Influenza virus NS1 protein interacts with viral transcription-replication complexes in vivo. J Gen Virol 78:2447–2451. doi:10.1099/0022-1317-78-10-2447. 9349463

[91] Zürcher T, Marión RM, Ortín J 2000 Protein synthesis shut-off induced by influenza virus infection is independent of PKR activity. J Virol 74:8781–8784. doi:10.1128/JVI.74.18.8781-8784.2000. 10954584 PMC116394

[92] Kawakami E, Watanabe T, Fujii K, Goto H, Watanabe S, Noda T, Kawaoka Y 2011 Strand-specific real-time RT-PCR for distinguishing influenza vRNA, cRNA, and mRNA. J Virol Methods 173:1–6. doi:10.1016/j.jviromet.2010.12.014. 21185869 PMC3049850

[93] Kim D, Pertea G, Trapnell C, Pimentel H, Kelley R, Salzberg SL 2013 TopHat2: accurate alignment of transcriptomes in the presence of insertions, deletions and gene fusions. Genome Biol 14:R36. doi:10.1186/gb-2013-14-4-r36. 23618408 PMC4053844

[94] Anders S, Pyl PT, Huber W 2015 HTSeq—a python framework to work with high-throughput sequencing data. Bioinformatics 31:166–169. doi:10.1093/bioinformatics/btu638. 25260700 PMC4287950

[95] Love MI, Huber W, Anders S 2014 Moderated estimation of fold change and dispersion for RNA-seq data with DESeq2. Genome Biol 15:550. doi:10.1186/s13059-014-0550-8. 25516281 PMC4302049

[96] Benjamini Y, Hochberg Y 1995 Controlling the false discovery rate: a practical and powerful approach to multiple testing. J R Stat Soc 57:289–300.

[97] Oliveros JC 2007 FIESTA@BioinfoGP: an interactive server for analyzing DNA microarray experiments with replicates. Centro Nacional de Biotecnología, Madrid, Spain http://bioinfogp.cnb.csic.es/tools/FIESTA/index.php.

