# Supplementary material for: Chemical Genomics Identifies the PERK-Mediated Unfolded Protein Stress Response as a Cellular Target for Influenza Virus Inhibition
Source: mBio. 2016 Apr 19;7(2):e00085-16. doi: 10.1128/mBio.00085-16 (PMC4850254; doi:10.1128/mBio.00085-16)
Supplement: Figure S1 — Screening parameters. The average values determined for 96 GFP-positive wells (μpos), negative wells (μneg), and the corresponding standard deviations (δpos and δneg) are shown. These values were used to calculate the reproducibility (Z), percent coefficient of variation (%CV), signal-to-background (S/B) ratio, and signal-to-noise (S/N) ratio with the formulas shown. Download [file mbo002162776sf1.pdf]

**μ_{pos}**	$\Sigma(n_{pos1}+n_{pos2}+\ldots+n_{posn})/n_{pos}$	**5,3x10^7**
**μ_{neg}**	$\Sigma(n_{neg1}+n_{neg2}+\ldots+n_{negn})/n_{neg}$	**4,68x10^3**
**δ_{pos}**	$[\Sigma(\mu_{pos})^2/(n-1)]^{1/2}$	**9,4x10^5**
**δ_{neg}**	$[\Sigma(\mu_{neg})^2/(n-1)]^{1/2}$	**7,36x10^2**
**Z**	$1-((3\delta_{pos}+3\delta_{neg})/(\mu_{pos}-\mu_{neg})$	**0,94**
**%CV**	δ_{pos}/μ_{pos} 100	**1,74%**
**S/B**	μ_{pos}/μ_{neg}	**1,15x10^4**
**S/N**	$(\mu_{pos}-\mu_{neg})/((\delta_{pos})^2+(\delta_{neg})^2)^{1/2}$	**7,32x10^4**

Supplementary Fig. S1. Screening parameters. The average values determined for 96 GFP positive wells (μ_{pos}), negative wells (μ_{neg}) and the corresponding standard deviations (δ_{pos} and δ_{neg}) are shown. These values were used to calculate the reproducibility (Z), variability coefficient (%CV), signal-to-background ratio (S/B) and signal-to-noise ratio (S/N) using the formulas specified in the Figure.
