# Supplementary material for: Chemical Genomics Identifies the PERK-Mediated Unfolded Protein Stress Response as a Cellular Target for Influenza Virus Inhibition
Source: mBio. 2016 Apr 19;7(2):e00085-16. doi: 10.1128/mBio.00085-16 (PMC4850254; doi:10.1128/mBio.00085-16)
Supplement: Figure S2 — Characterization of the MK preparation used for screening. (A) Chemical structure of MK obtained from the PubChem database (CID 23663996) corresponding to the formula C35H35ClNNaO3S and a molecular mass of 608.16 g/mol. The mass spectra of samples of the compound MK obtained from the NIH library (B) and AKOS GmbH (C), as determined by matrix-assisted laser desorption ionization--time of flight tandem mass spectrometry, are shown. Peaks correspond to ionized fragments of the compound generated by laser irradiation and separated by mass/charge ratios with a mass spectrometer. Download [file mbo002162776sf2.pdf]

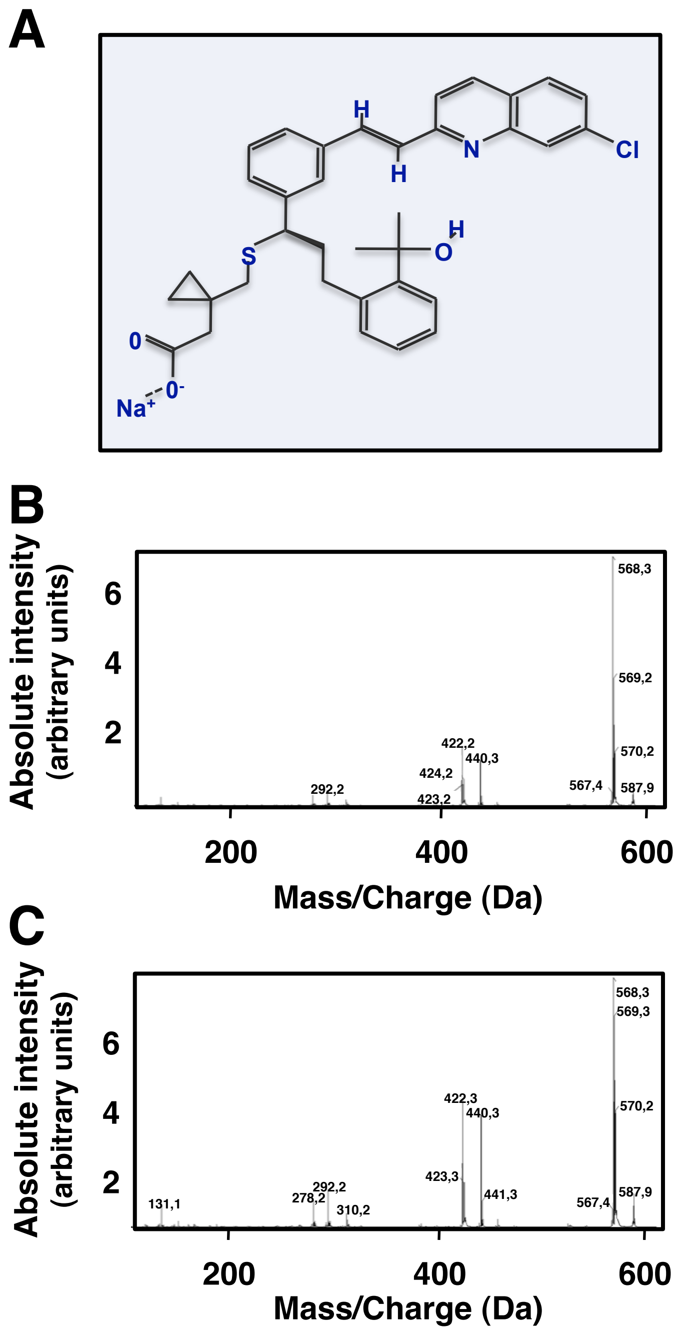

Supplementary Fig. S2. Characterisation of the Montelukast preparation used for screening. (A) Chemical structure of Montelukast obtained from PubChem database (CID 23663996) corresponding to the formula $C_{35}H_{35}ClNNaO_3S$ and the molecular weight of 608.16 g/mol. Mass spectrum of the compound Montelukast obtained from NIH library (B) and from AKOS GMBH (C) by MALDI-TOF/TOF. Peaks correspond to ionized fragments of the compound generated by laser irradiation and separated by mass/charge using a mass spectrometer.
