# Supplementary material for: Chemical Genomics Identifies the PERK-Mediated Unfolded Protein Stress Response as a Cellular Target for Influenza Virus Inhibition
Source: mBio. 2016 Apr 19;7(2):e00085-16. doi: 10.1128/mBio.00085-16 (PMC4850254; doi:10.1128/mBio.00085-16)
Supplement: Figure S3 — Splicing of influenza virus mRNA is not altered by MK treatment. Cultures of human A549 cells were infected with influenza virus at an MOI of 3 PFU/cell and then treated with 20 µM MK or the corresponding amount of DMSO. Total cell RNA was isolated at 6 hpi, and rRNA was removed. Viral RNA of each segment was determined by deep sequencing and classified according to the virus segment and polarity. The splicing efficiencies of segments 7 (M2/total M) and 8 (NEP/total NS) are presented as reads in splice junctions versus the total reads in each segment mRNA. Download [file mbo002162776sf3.pdf]

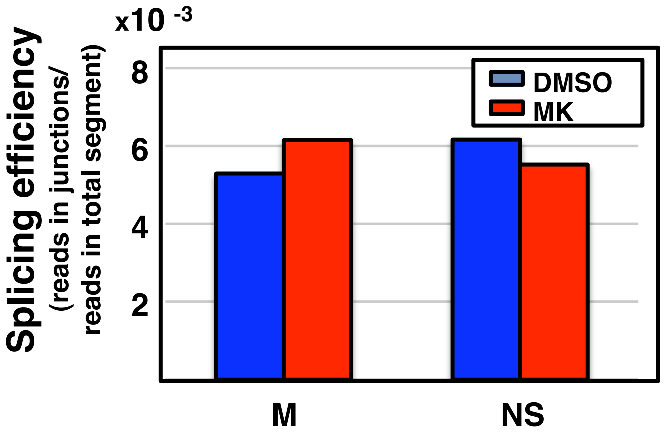

Supplementary Fig. S3. Splicing of influenza virus mRNA is not altered by Montelukast treatment. Cultures of human A549 cells were infected with influenza virus at a moi of 3 pfu/cell and then treated with 20 μM of Montelukast or the corresponding amount of DMSO. Total cell RNA was isolated at 6 hpi and ribosomal RNA was removed. Viral RNA of each segment was determined by deep-sequencing and classified according to virus segment and polarity. Splicing efficiency of segment 7 (M2 / total M) and 8 (NEP / total NS) are presented as reads in splice junctions versus total reads in each segment mRNA.
