# Supplementary material for: Chemical Genomics Identifies the PERK-Mediated Unfolded Protein Stress Response as a Cellular Target for Influenza Virus Inhibition
Source: mBio. 2016 Apr 19;7(2):e00085-16. doi: 10.1128/mBio.00085-16 (PMC4850254; doi:10.1128/mBio.00085-16)
Supplement: Figure S4 — Functional protein association network of the genes differentially expressed in influenza virus-infected cells upon treatment with MK. Shown is the protein-protein interaction network as visualized by the STRING database. The nodes represent proteins, red circles contain most of the proteins differentially expressed in influenza virus-infected cells upon treatment with MK, and the lines represent the predicted functional associations. The associations were inferred from several types of evidence from the STRING database: the presence of experimental evidence (pink line), text mining evidence from abstracts of the scientific literature (yellow line), information from databases (light blue), sequence homology (light gray), co-occurrence evidence (dark blue), and coexpression evidence (dark gray). Retrieved from STRING 9.1, 2015. Download [file mbo002162776sf4.pdf]

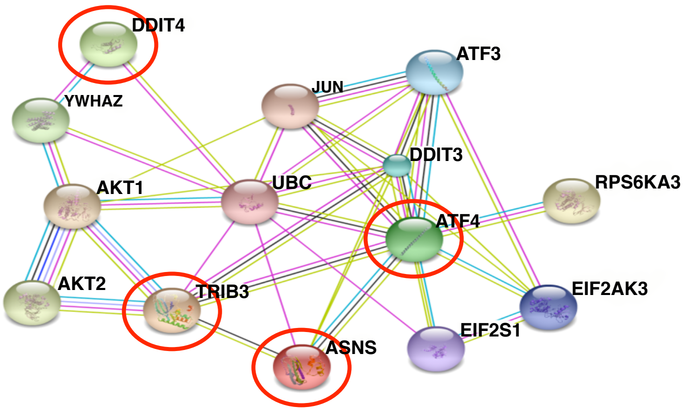

Supplementary Fig. S4. Functional protein association network of the genes differentially expressed in influenza virus-infected cells upon treatment with Montelukast. Protein-protein interaction network as visualized by STRING database. The nodes represent proteins; red circles contained most of the proteins differentially expressed in influenza virus-infected cells upon treatment with Montelukast; the lines represent the predicted functional associations. The associations were inferred from several types of evidence from the STRING database: the presence of experimental evidence (pink line), text-mining evidence from abstracts of scientific literature (yellow line), information from databases (light blue), sequence homology (light grey), co-occurrence evidence (dark blue) or co-expression evidence (dark grey). Retrieved from STRING 9.1, 2015.

SUPP FIGURE 4
