# Supplementary material for: Chemical Genomics Identifies the PERK-Mediated Unfolded Protein Stress Response as a Cellular Target for Influenza Virus Inhibition
Source: mBio. 2016 Apr 19;7(2):e00085-16. doi: 10.1128/mBio.00085-16 (PMC4850254; doi:10.1128/mBio.00085-16)
Supplement: Figure S5 — MK inhibits viral protein synthesis. Cultures of human A549 cells were infected with the New Caledonia (H1N1) or VIC (H3N2) strain of influenza virus or with VSV at an MOI of 3 PFU/cell and then treated with 40 µM MK or the corresponding amount of DMSO. At 6 hpi, the cultures were pulse-labeled with [35S]methionine-cysteine and total protein extracts were prepared. (A) The samples were analyzed by polyacrylamide gel electrophoresis and autoradiography. The mobility of some of the virus-specific proteins (stars for influenza virus and arrows for VSV) is indicated to the right. (B) Quantification of the signals of DMSO-treated (blue) and MK-treated (red) samples. Download [file mbo002162776sf5.pdf]

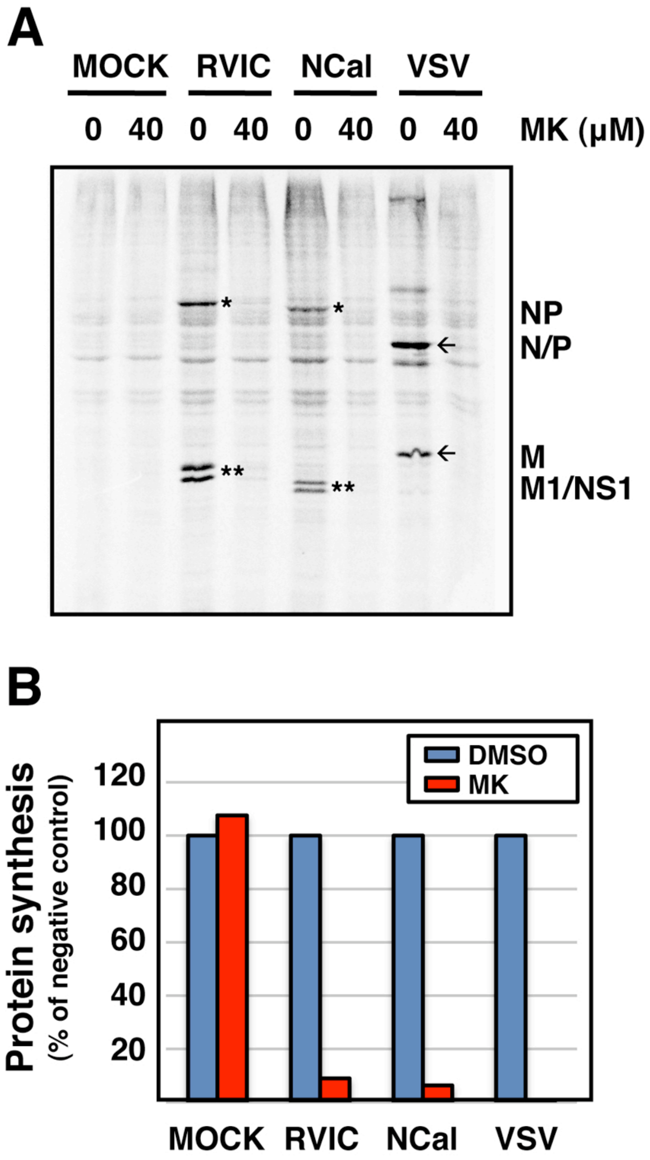

Supplemental Fig. S5. Montelukast inhibits viral protein synthesis. Cultures of human A549 cells were infected with the New Caledonian (H1N1) or the Victoria (H3N2) strains of influenza virus or with Vesicular Stomatitis virus (VSV) at a moi of 3 pfu/cell and then treated with 40 µM of Montelukast or the corresponding amount of DMSO. At 6 hpi the cultures were pulse-labelled with ^{35}S-met-cys and total protein extracts were prepared. (A) The samples were analysed by polyacrylamide gel electrophoresis and autoradiography. The mobility of some of the virus specific proteins (stars for influenza virus and arrows for VSV) is indicated to the right. (B) Quantification of the signals of DMSO-treated (blue) or MK-treated samples (red).
