# Supplementary material for: Chemical Genomics Identifies the PERK-Mediated Unfolded Protein Stress Response as a Cellular Target for Influenza Virus Inhibition
Source: mBio. 2016 Apr 19;7(2):e00085-16. doi: 10.1128/mBio.00085-16 (PMC4850254; doi:10.1128/mBio.00085-16)
Supplement: Table S1 — Alterations of the ATF6- and IRE1-dependent unfolded protein response pathways by influenza virus infection (FLU) and/or MK treatment. Shown are the genes regulated by the transcription factors ATF6 and IRE1. The identification (ID) code in the Ensembl database is specified, as well as the FC and the FDR. [file mbo002162776st1.pdf]

			FLU-MK vs FLU		MOCK-MK vs MOCK		FLU vs MOCK	
RECEPTOR	GENE	ID	FC	FDR	FC	FDR	FC	FDR
IRE1	EDEM	ENSG00000134109	0.92	0.99	1.02	0.99	1.04	0.76
	HEDJ	ENSG00000090520	1.04	0.99	1.11	0.99	1.12	0.39
	RAMP4	ENSG00000120742	1.04	0.99	-1.09	0.99	1.28	0.5×10^{-2}
	p58IPK	ENSG00000102580	0.97	0.99	-1.06	0.99	-1.47	1.1×10^{-5}
	ERdj4	ENSG00000128590	0.95	0.99	1.04	0.99	1.12	0.53
	XBP1	ENSG00000100219	1.21	0.83	1.34	0.39	1.10	0.39
ATF6	HERP	ENSG00000051108	1.37	0.99	1.56	0.02	-1.37	0.027
	CREB4	ENSG00000143578	1.13	0.99	1.16	0.99	-1.23	0.52
	LUMAN	ENSG00000164463	-1.04	0.99	1.23	0.99	2.82	$<1 \times 10^{-8}$
	BBF2H7	ENSG00000182158	-1.14	0.99	-1.21	0.99	-1.87	$<1 \times 10^{-8}$

Supplementary Table S1. Alterations of the ATF6- and IRE1-dependent unfolded protein response pathways by virus infection and/or Montelukast treatment. The Table shows the genes regulated by the transcription factors ATF6 and IRE1. The identification code of Ensembl database (ID) is specified as well as the fold change (FC) and the false discovery rate (FDR).
