# Supplementary material for: Chemical Genomics Identifies the PERK-Mediated Unfolded Protein Stress Response as a Cellular Target for Influenza Virus Inhibition
Source: mBio. 2016 Apr 19;7(2):e00085-16. doi: 10.1128/mBio.00085-16 (PMC4850254; doi:10.1128/mBio.00085-16)
Supplement: Table S2 — Lack of alteration of genes downregulated by virus infection upon MK treatment. Shown are the genes downregulated by influenza virus infection (FLU versus MOCK; cutoff values, FC of less than −3 and FDR of <10−3) and the lack of their modification by treatment with MK (FLU-MK versus FLU, MOCK-MK versus MOCK). The identification (ID) code in the Ensembl database is specified, as well as the FC and the FDR. [file mbo002162776st2.pdf]

		FLU-MK vs FLU		MOCK-MK vs MOCK		FLU vs MOCK	
**GENE**	**ID**	**FC**	**FDR**	**FC**	**FDR**	**FC**	**FDR**
**KCNK5**	ENSG00000164626	1.06	0.99	0.95	0.99	-4.11	8.9×10^{-7}
**MYH9**	ENSG00000100345	1.01	0.99	1.09	0.99	-3.56	$<1 \times 10^{-8}$
**CD97**	ENSG00000123146	1.25	0.99	0.86	0.99	-3.46	$<1 \times 10^{-8}$
**SUN2**	ENSG00000100242	1.27	0.99	1.27	0.99	-3.43	$<1 \times 10^{-8}$
**KLHDC7A**	ENSG00000179023	1.16	0.99	0.99	0.99	-3.41	8.8×10^{-4}
**LAMB2**	ENSG00000172037	1.27	0.99	1.04	0.99	-3.39	$<1 \times 10^{-8}$
**FZD2**	ENSG00000180340	1.51	0.99	0.93	0.99	-3.32	1.6×10^{-5}
**FBN2**	ENSG00000138829	0.98	0.99	1.06	0.99	-3.27	$<1 \times 10^{-8}$
**IBA57**	ENSG00000181873	1.18	0.99	0.91	0.99	-3.27	6.1×10^{-6}
**NCDN**	ENSG00000020129	1.24	0.99	1.04	0.99	-3.25	0.2×10^{-4}
**LRRC8E**	ENSG00000171017	1.06	0.99	0.85	0.99	-3.25	4.3×10^{-5}
**MUC5AC**	ENSG00000215182	1.67	0.99	1.22	0.99	-3.25	$<1 \times 10^{-8}$
**FASN**	ENSG00000169710	1.38	0.99	1.16	0.99	-3.16	$<1 \times 10^{-8}$
**SLC29A3**	ENSG00000198246	1.16	0.99	0.94	0.99	-3.16	3.9×10^{-4}
**PLEKHG4**	ENSG00000196155	1.36	0.99	1.16	0.99	-3.14	8.1×10^{-6}

Supplemental Table S2. Lack of alteration of genes down-regulated by virus infection upon Montelukast treatment. The Table shows the genes down-regulated by influenza virus infection (Flu vs Mock. Cut-off values FC<-3; FDR<10^{-3}) and the lack of their modification by treatment with MK (Flu-MK vs Flu; Mock-MK vs Mock). The identification code of Ensembl database (ID) is specified as well as the fold change (FC) and the false discovery rate (FDR).
