# Supplementary material for: Chemical Genomics Identifies the PERK-Mediated Unfolded Protein Stress Response as a Cellular Target for Influenza Virus Inhibition
Source: mBio. 2016 Apr 19;7(2):e00085-16. doi: 10.1128/mBio.00085-16 (PMC4850254; doi:10.1128/mBio.00085-16)
Supplement: Table S3 — Alterations of GADD34/PPP1R15α transcription by influenza virus infection (FLU) and/or MK treatment. The identification (ID) code in the Ensembl database is specified, as well as the FC and the FDR. [file mbo002162776st3.pdf]

		FLU-MK vs FLU		MOCK-MK vs MOCK		FLU vs MOCK	
**GENE**	**ID**	**FC**	**FDR**	**FC**	**FDR**	**FC**	**FDR**
**PPP1R15A**	ENSG00000087074	1.11	0.99	1.32	0.99	2.60	$<1 \times 10^{-8}$

Supplemental Table S3. Alterations of GADD34/PPP1R15a transcription by virus infection and/or Montelukast treatment. The identification code of Ensembl database (ID) is specified as well as the fold change (FC) and the false discovery rate (FDR).
